# Toxic stress-specific cytoprotective responses regulate learned behavioral decisions in *C. elegans*

**DOI:** 10.1186/s12915-021-00956-y

**Published:** 2021-02-09

**Authors:** Gábor Hajdú, Eszter Gecse, István Taisz, István Móra, Csaba Sőti

**Affiliations:** 1 Department of Molecular Biology, Semmelweis University, Budapest, Hungary; 2 Current Address: Neurobiology Division, MRC Laboratory of Molecular Biology, Cambridge, UK

**Keywords:** Physiological defense, Stress and detoxification responses, Inter-tissue signaling, Aversive behavior, Avoidance learning, Fight-or-flight response, Associative memory

## Abstract

**Background:**

Recognition of stress and mobilization of adequate “fight-or-flight” responses is key for survival and health. Previous studies have shown that exposure of *Caenorhabditis elegans* to pathogens or toxins simultaneously stimulates cellular stress and detoxification responses and aversive behavior. However, whether a coordinated regulation exists between cytoprotective stress responses and behavioral defenses remains unclear.

**Results:**

Here, we show that exposure of *C. elegans* to high concentrations of naturally attractive food-derived odors, benzaldehyde and diacetyl, induces toxicity and food avoidance behavior. Benzaldehyde preconditioning activates systemic cytoprotective stress responses involving DAF-16/FOXO, SKN-1/Nrf2, and Hsp90 in non-neuronal cells, which confer both physiological (increased survival) and behavioral tolerance (reduced food avoidance) to benzaldehyde exposure. Benzaldehyde preconditioning also elicits behavioral cross-tolerance to the structurally similar methyl-salicylate, but not to the structurally unrelated diacetyl. In contrast, diacetyl preconditioning augments diacetyl avoidance, weakens physiological diacetyl tolerance, and does not induce apparent molecular defenses. The inter-tissue connection between cellular and behavioral defenses is mediated by JNK-like stress-activated protein kinases and the neuropeptide Y receptor NPR-1. Reinforcement of the stressful experiences using spaced training forms stable stress-specific memories. Memory retrieval by the olfactory cues leads to avoidance of food contaminated by diacetyl and context-dependent behavioral decision to avoid benzaldehyde only if there is an alternative, food-indicative odor.

**Conclusions:**

Our study reveals a regulatory link between conserved cytoprotective stress responses and behavioral avoidance, which underlies “fight-or-flight” responses and facilitates self-protection in real and anticipated stresses. These findings imply that variations in the efficiency of physiological protection during past episodes of stress might shape current behavioral decisions.

**Graphical abstract:**

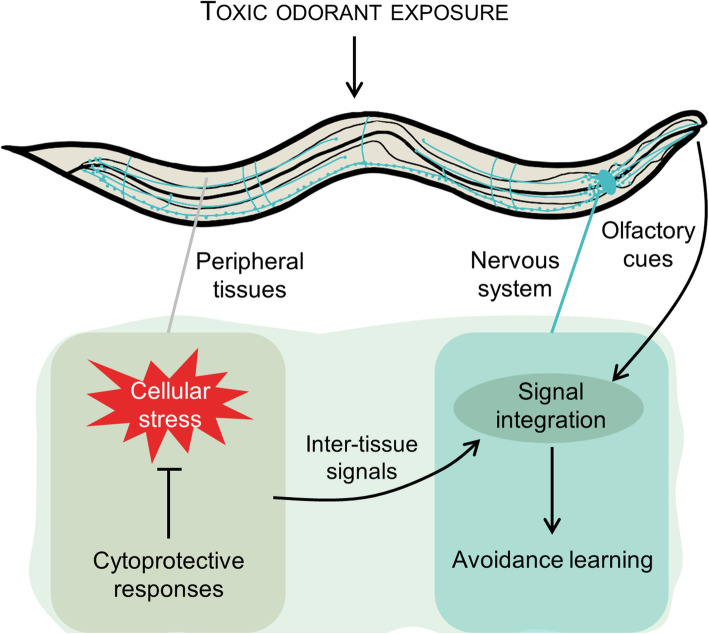

**Supplementary Information:**

The online version contains supplementary material available at 10.1186/s12915-021-00956-y.

## Background

Adequate, coordinated responses of multicellular organisms are key to adapt to and overcome fundamental alterations of the environment [1, 2]. These responses originate from intracellular molecular defenses, such as the oxidative, xenobiotic, metabolic, and proteotoxic stress responses, which guard homeostasis and confer cytoprotection against the respective stresses, promoting physiological adaptation, fitness, and longevity at the organismal level [3]. Adaptation also involves complex behavioral responses orchestrated by the neuroendocrine system [4]. For instance, sensory cues representing danger evoke aversive behavior as a result of the perception of multiple sensory stimuli, neuronal processing, and decision-making both in humans and in other species [5, 6]. In some cases, the neural impulse of perceived danger can evoke the avoidance of cues representing important resources, such as food [7]. Besides external sensory cues, decision-making is modulated by neural context like arousal, motivation, and reward [8]. Importantly, behavioral decisions are also influenced by sensory cues that evoke associative memories of past events [9]. Moreover, exaggerated avoidant behavior is characteristic of human anxiety disorders such as phobias [7], where sometimes intense physical symptoms of toxicity and disgust are evoked by olfactory or gustatory cues. Although the neuroendocrine mechanisms of stress are extensively studied, the contribution of intracellular defenses to behavioral regulation is largely unknown.

The nematode *Caenorhabditis elegans* with its 959 cells is a versatile model system to study the link between cytoprotective stress responses and behavior. Worms, using a well-defined network of 302 neurons, are capable of complex behavioral decisions [5, 10, 11]. Odors and flavors have a great impact on the decision-making of nematodes, informing about possible nutrition or danger via neuronal processing of olfactory and gustatory cues, resulting in attraction or aversion [11]. Besides well-characterized escape responses, tissue-damaging insults, such as toxins and pathogens, induce a network of evolutionary conserved cytoprotective defenses in each cell and in specialized tissues [3]. Fixing the actual damage and eliminating damaging agents are key mechanisms of cellular protection [12]. Nematodes and mammals share diverse molecular processes to recognize and overcome toxic, stressor agents, such as the FOXO and Nrf2 pathways. A key oxidative and metabolic stress response regulator in *C. elegans* is the FOXO ortholog DAF-16 transcription factor. DAF-16 is ubiquitously expressed, localized in the cytosol, and is activated by nuclear translocation in response to oxidative and genotoxic agents, starvation, desiccation, and heat stress [13]. Loss-of-function mutations or RNAi knockdown of *daf-16* results in compromised resistance to multiple stresses and shorter lifespan [14].

The Nrf2 ortholog SKN-1 transcription factor is the major xenobiotic and oxidative stress regulator in nematodes [12]. Its nuclear translocation is induced by dietary restriction, pathogen attack, the INS/IGF-1 and TIR-1/PMK-1 pathways to modulate cellular respiration, enhance oxidative stress resistance, immunity, and systemic detoxification defenses [15, 16]. SKN-1 cooperates with numerous stress-related pathways and regulators including DAF-16 and the *C. elegans* heat shock transcription factor ortholog HSF-1 to fine-tune cytoprotective gene expression patterns [12]. Upregulation of specific and overlapping molecular stress responses underlies an adaptive process called “physiological conditioning hormesis” in stress biology [17]. In the course of hormesis, a conditioning (or preconditioning) physical stress exposure results in increased survival upon a subsequent, lethal stress evoked by the same or a different stressor, a phenomenon called stress tolerance or cross-tolerance, respectively. However, a conditioning exposure might also cause distress and decreased protection against a subsequent lethal stress in the absence of sufficient physiological stress responses. In behavioral science, conditioning or training means a learning process elicited by a biologically relevant stimulus. To clearly discriminate physiological and behavioral terms, we use the term “preconditioning” for physiological conditioning to emphasize the induction of physiological stress responses and introduce the term “behavioral tolerance” for the diminished avoidance of the stimulus after a preconditioning stress.

Recent studies in *C. elegans*, including ours, provided evidence that pathogen- and toxin-induced stresses simultaneously stimulate cytoprotective responses and aversive behavior [18–20]. In this study, we set out to investigate how the induction of systemic cytoprotective molecular defenses influences stress-induced aversive behavior and learned behavioral decisions. To this end, we employed two food-derived volatile odorants, benzaldehyde (BA) and diacetyl (DA), which are attractive at low, but aversive at high concentrations [21, 22]. The advantage of these odors is that they contain both the chemosensory cue as well as a dual, attractive, or aversive property. Our results suggest that the ability to mount stress-specific cytoprotective responses in non-neuronal cells shapes adaptive stress-induced and subsequent behavioral decisions through the modulation of avoidance learning.

## Results

### Undiluted benzaldehyde and diacetyl induce food avoidance behavior and toxicity

Low concentrations of food odors are attractive to *C. elegans*, whereas high concentrations induce an aversive response [22]. Specifically, worms exhibit a biphasic chemotaxis curve towards undiluted 100% benzaldehyde called benzotaxis [21]. (Throughout the study, we refer to diluted benzaldehyde as BA, and to the undiluted volatiles using the “cc” *concentratus* prefix, e.g., undiluted benzaldehyde as ccBA). The exclusive preservation of avoidance in the *odr-3* chemosensory mutant that mediates attraction to low concentrations of BA, and its sensitivity to dishabituation suggested that aversion is an independent behavior which appeared after habituation to the attractive stimulus in the absence of food [21]. We confirmed the biphasic behavior in kinetic chemotaxis experiments (Additional File 1: Fig. S1a). However, the same 30-min lag phase preceding aversion in both wild-type and “genetically habituated” *odr-3* nematodes (*29* and Additional File 1: Fig. S1a) suggested that animals might develop the second, aversive phase independently of habituation and only after sufficient exposure to the undiluted odor.

**Fig. 1:**
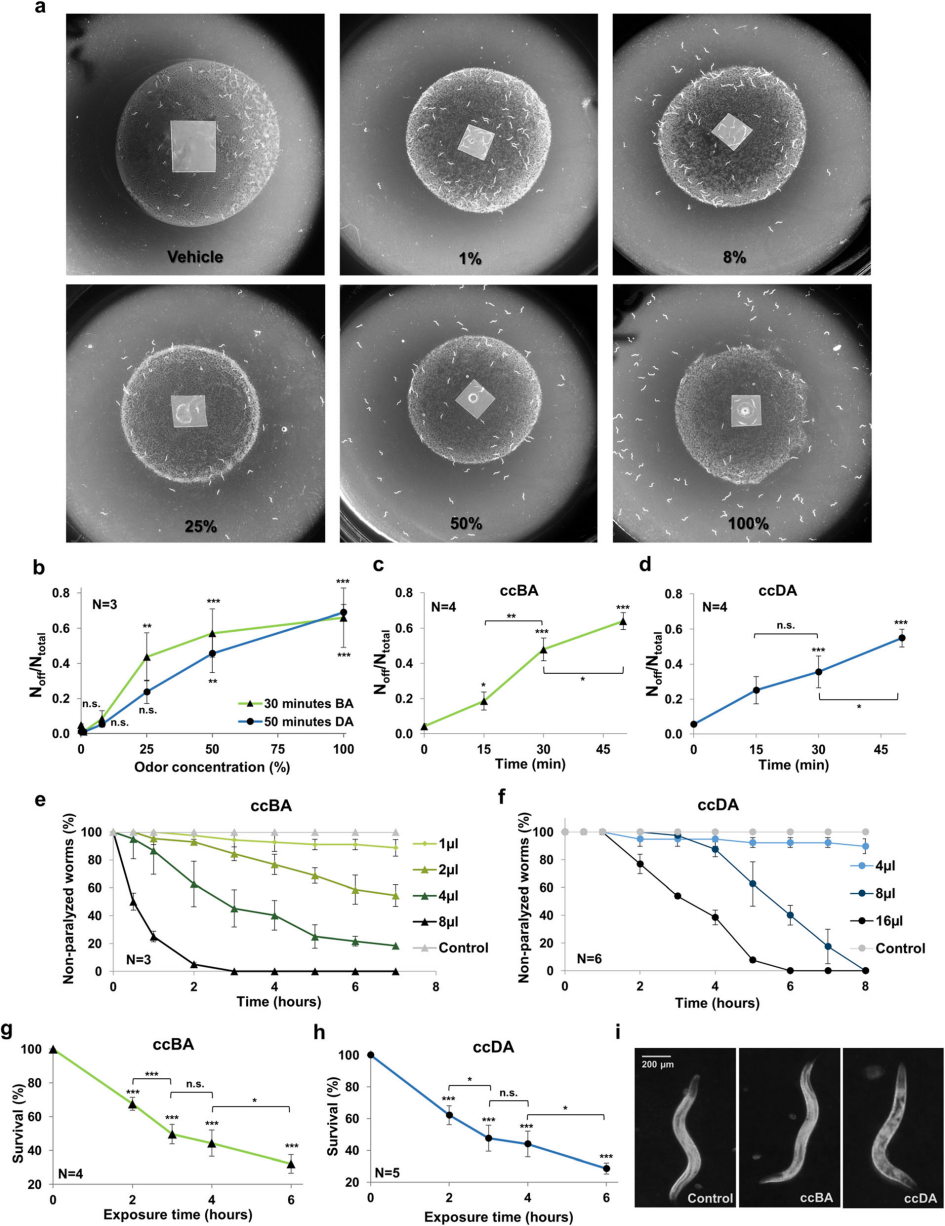
Undiluted benzaldehyde (ccBA) and diacetyl (ccDA) induce food aversion and toxicity. **a** Representative images of food leaving behavior in response to a 50-min exposure to various concentrations of BA. BA was placed in an ethanol vehicle in a total volume of 1 μl in the middle of the bacterial lawn. **b** Dose dependence of food avoidance after a 30-min exposure to BA or a 50-min exposure to DA. BA or DA was placed in a total volume of 1 μl or 4 μl in the middle of the bacterial lawn. **c** Time dependence of 1 μl ccBA-induced food avoidance. **d** Time dependence of 4 μl ccDA-induced food avoidance. **e** Time dependence curves of paralysis to various doses of ccBA. **f** Time dependence curves of paralysis to various doses of ccDA. **g** Exposure time dependence of survival to the highest, 8 μl dose of ccBA. **h** Exposure time dependence of survival to the highest, 16 μl dose of ccDA. Survival was scored 14 h after the end of exposures. **i** Representative stereomicroscopic images of worms 14 h after a 3-h exposure to 8 μl ccBA or 16 μl ccDA. The mean durations of odor exposure that induced 50% paralysis by log rank (Mantel-Cox) test were as follows: ccBA - 2 μl 5.27 h ± 0.17 h, 4 μl 2.94 ± 0.21 h, and 8 μl 0.94 ± 0.14 h; ccDA - 8 μl 5.68 ± 0.20 h and 16 μl 3.46 ± 0.17 h. Compared to 1 μl BA or 4 μl DA treatments *p* < 0.001 in all conditions. Data are expressed as mean ± SEM. *N*, number of independent experiments. *p* values were obtained by one-way ANOVA with Fisher’s LSD post hoc test. n.s., not significant; *p < 0.05; **p < 0.01; ***p < 0.001

This phenomenon is reminiscent of behavioral avoidance elicited by noxious stimuli. Indeed, worms are continuously feeding on nutritious bacteria under laboratory conditions, but they leave pathogen- and toxin-contaminated bacterial lawns [18, 23]. We hypothesized that if aversion is a defensive behavioral response and is independent of habituation and/or olfactory adaptation, then ccBA will also trigger nematodes to leave the food lawn rich in chemosensory and nutritive stimuli. To investigate this possibility, we placed a ccBA drop on a parafilm in the middle of a central *Escherichia coli* OP50 lawn, where worms acclimatized for 30 min and monitored food avoidance. Using a ccBA dose proportionally considering the plate volume used in kinetic chemotaxis experiments, we observed that while mock-exposed worms remained on the lawn after 50 min, the majority of the ccBA-exposed worms left the food (Fig. 1a). Diacetyl (DA), a chemically unrelated food odor, is also aversive at high concentrations [22] and also triggered a biphasic chemotaxis behavior (Additional File 1: Fig. S1b). We found that both ccBA and ccDA elicited concentration-dependent food aversion phenotypes (Fig. 1b). Further, we observed a time-dependent development of food aversion for both volatiles (Fig. 1c, d), which, even though food was present, showed a faster kinetics, than that in the kinetic chemotaxis experiments. In contrast to the aversive effect of undiluted odors, only a negligible fraction of worms left the lawn containing vehicle, whereas they entirely remained on the lawn in the presence of attractive, 1% concentrations of BA or DA (Fig. 1a, b and Additional File 1: Fig. S1c). As starvation induces both adaptation and habituation [24], both neuronal mechanisms to the undiluted odors might occur in the absence of food. However, worms not only decreased their sensory perception of, or their interest towards, inconsequential odors but actively vacated the lawn to reach the furthest possible distance from the odor source. Taken together, giving up the advantage of nutrition is a consequence of a defensive behavioral decision to avoid a harmful stimulus.

To address if animals avoided ccBA and ccDA because of toxic effects, we evaluated the paralysis rate of worms subjected to different undiluted odor doses. We found that longer ccBA and ccDA exposures to higher doses induced extensive paralysis in a dose- and time-dependent manner (Fig. 1e, f). Then, we estimated toxicity by monitoring survival [25] the day after exposure to the highest doses of the respective undiluted odors and observed that ccBA and ccDA similarly induced death in an exposure time-dependent manner (Fig. 1g, h). Accordingly, we detected a marked deterioration of the internal structure of animals after the exposure to the highest dose of ccDA, compared to a preserved morphology after that of ccBA (Fig. 1i). Importantly, extended exposure to doses of ccBA and ccDA used in food leaving assays was not apparently toxic per se (Fig. 1e, f), but both impaired thermotolerance (i.e., the ability to withstand heat stress) (Additional File 1: Fig. S1d). The impaired stress tolerance, paralysis, and death by increasing doses of ccBA and ccDA represent a progressive disruption of physiological homeostasis. Based on these findings, we hypothesized that the behavioral avoidance of the undiluted odorants may be a consequence of their toxic effect.

### Opposing behavioral and physiological outcomes elicited by toxic benzaldehyde and diacetyl exposure

**Fig. 2:**
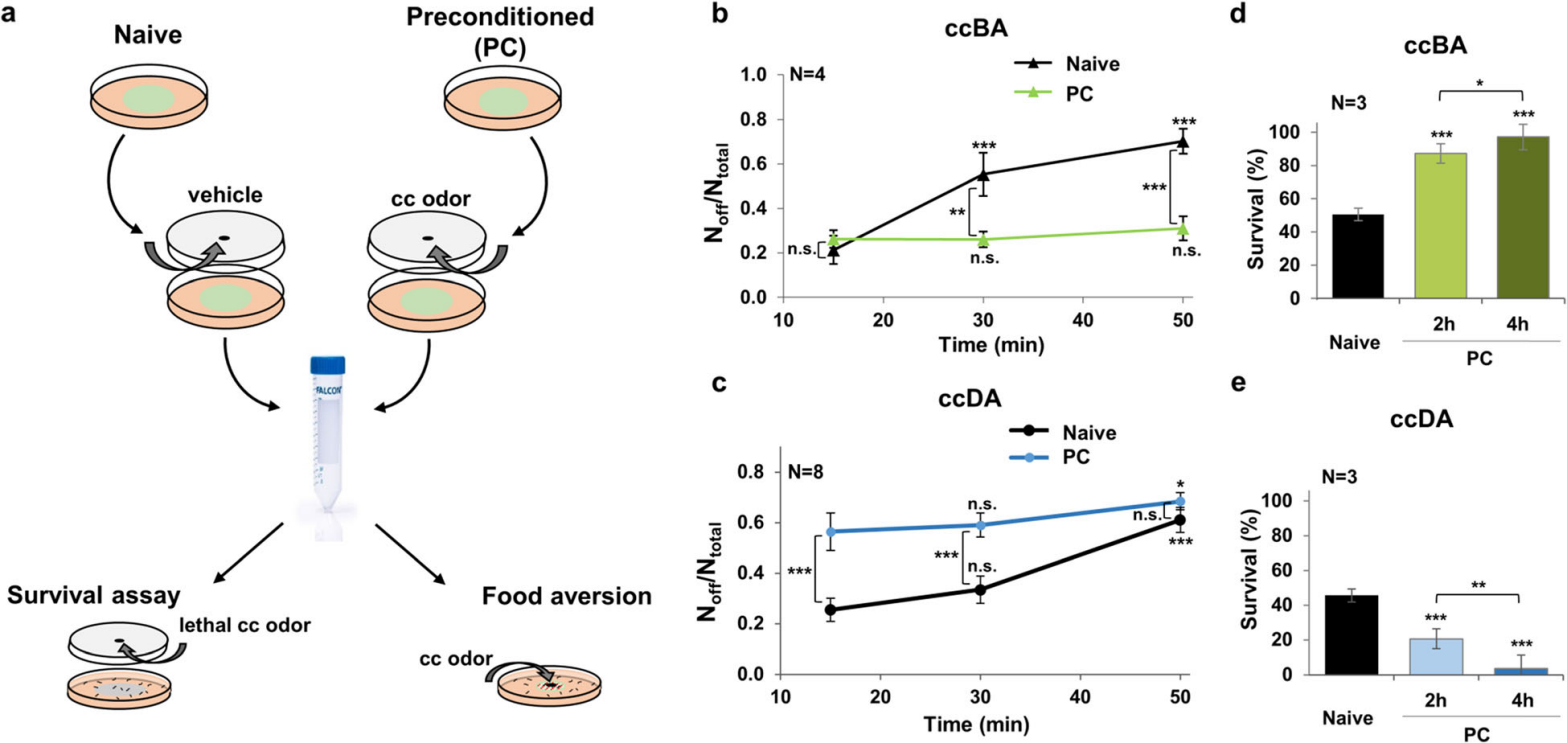
ccBA preconditioning induces behavioral and physiological stress tolerance, while ccDA preconditioning induces sensitization. **a** Experimental setup for preconditioning, followed by food aversion and survival tests. Animals were exposed to a hanging drop of undiluted odor (preconditioned, PC) or vehicle (naive), washed, and assayed for food avoidance or survival by exposure to the same or a lethal odor dose, respectively. **b** Food aversion induced by 1 μl ccBA of naive and ccBA-preconditioned (1 μl for 4 h) animals at different time points. **c** Food aversion induced by 4 μl of ccDA of naive and ccDA-preconditioned (4 μl for 4 h) animals at different time points. **d** Survival of naive and ccBA-preconditioned worms 14 h after a 3-h exposure to 8 μl ccBA. **e** Survival of naive and ccDA-preconditioned worms 14 h after a 3-h exposure to 16 μl ccDA. Data are expressed as mean ± SEM. *N*, number of independent experiments. *p* values were obtained by one-way ANOVA with Fisher’s LSD post hoc test. n.s., not significant; *p < 0.05; **p < 0.01; ***p < 0.001

We observed that transient exposure to higher doses of ccBA and ccDA increased motility (Additional File 1: Fig. S2a), suggesting that perception of toxic stress increases locomotor activity which may help instantly escape from the threat. Interestingly, the increased motility returned to baseline after removing ccBA but showed a sustained elevation after the removal of ccDA (Additional File 1: Fig. S2a). Moreover, we found that after an extended 2-h exposure to ccBA, animals started to return to the bacterial lawn, whereas the same exposure to ccDA further increased aversion (Additional File 1: Fig. S2b). Thus, the adverse physiological effects of ccBA might be eliminated faster than those of ccDA. We reasoned that a preconditioning exposure might differentially affect the defensive behavior to ccBA and to ccDA. To test this, after exposure, we preconditioned the worms by exposing them to the same doses of odors for 4 h on a large bacterial food lawn. After washing, we placed them on a small lawn and monitored their lawn avoidance behavior (Fig. 2a). We found that preconditioning with ccBA largely diminished ccBA-induced aversion for the entire duration of the experiment. In contrast, preconditioning with ccDA robustly increased the speed of ccDA lawn avoidance, reaching almost maximal value within 15 min (Fig. 2b, c). We tested whether the rapid food leaving might be due to an uncontrolled hypermotility observed at the two-fold ccDA dose. However, ccDA in the dose used in the food leaving assays for 30 min or for the entire 4-h preconditioning period did not augment, rather decreased worms’ motility (Additional File 1: Fig. S2c). Thus, the fast ccDA lawn avoidance, despite the reduced motility, appears to be the consequence of a directed navigation away from a familiar noxious stimulus. Preconditioning-induced behavioral changes were apparent at 2 h and were most pronounced at 4 h of pre-exposure (Additional File 1: Fig. S2d, e).

For the increased capacity of worms to remain in the presence of toxic ccBA, we used the term “behavioral tolerance,” to the analogy of physiological stress tolerance (i.e., the capacity to resist physical stress by engaging physiological defenses, such as cytoprotective stress responses). To investigate whether the contrasting behavioral responses evoked by the two volatiles were accompanied by similar outcomes in physiological stress tolerance, we preconditioned the worms with the lower, non-toxic odor doses used in the food leaving assays, then subjected them to lethal odor doses for 3 h and evaluated their survival 14 h after the end of the exposure. With increasing preconditioning time, we observed a robust survival increase on ccBA and a complete survival decline on ccDA (Fig. 2d, e), representing a protective (hormetic) effect of ccBA and a debilitating (distressing) effect of ccDA preconditioning. Hormesis and distress are well-known phenomena in stress biology and suggest efficient or insufficient physiological responses to the stress induced by ccBA or ccDA exposures, respectively [17]. These findings are consistent with those on Fig. 1, i.e., similar survival rates of animals on the respective odors, showing a recovery of ccBA-exposed worms from a transient early paralysis compared to the progressive decline after modest initial paralysis of ccDA-exposed worms (cf. Fig. 1e–h, 2–4 h of exposure). Thus, ccBA preconditioning induces behavioral and physiological stress tolerance, while ccDA preconditioning induces behavioral sensitization and physiological distress. These results suggest that nematodes can mount efficient physiological protection against ccBA, but can only engage more alert behavioral defense through sensitization against ccDA.

### Undiluted benzaldehyde, but not diacetyl, activates specific systemic cytoprotective responses

**Fig. 3:**
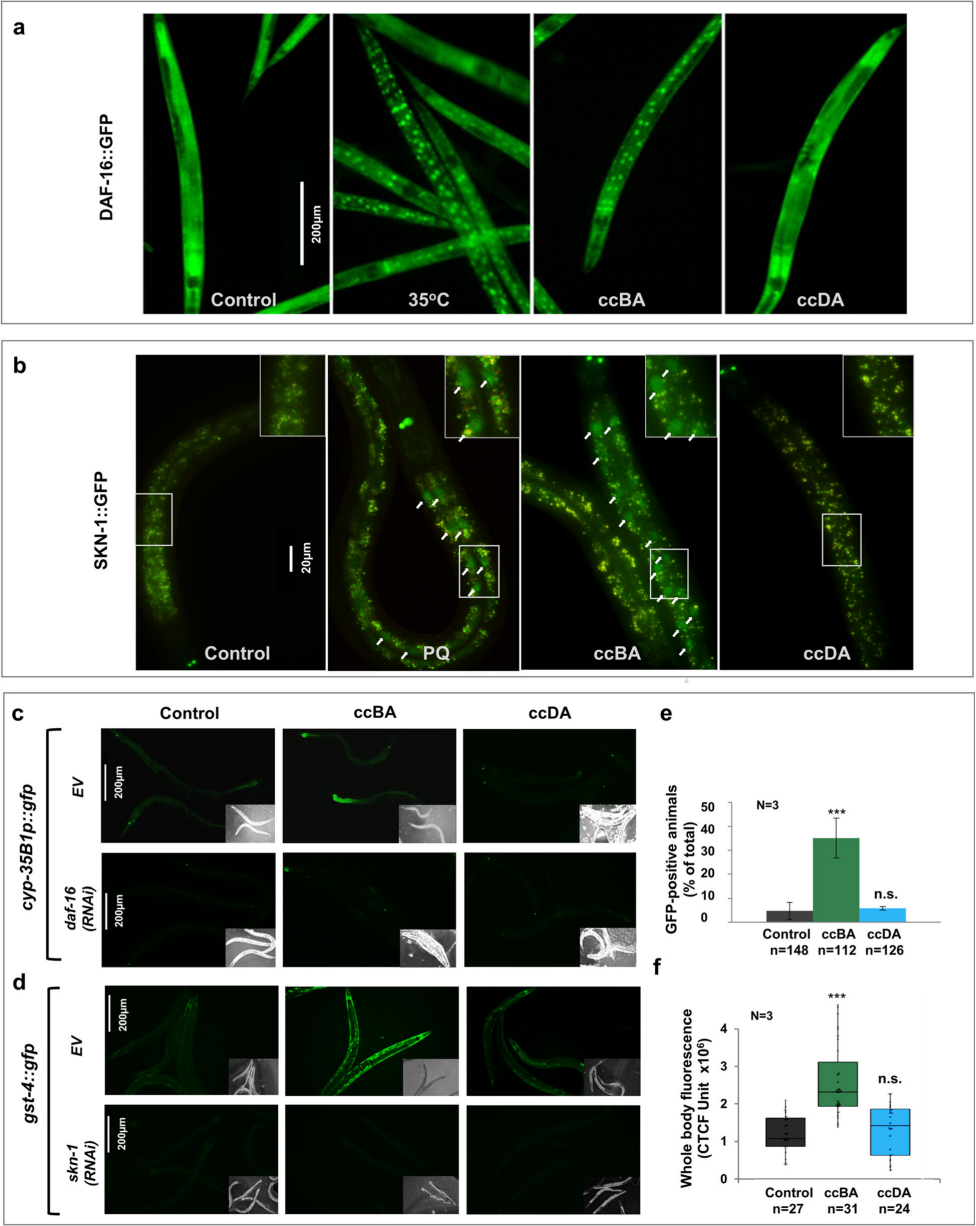
ccBA, but not ccDA, activates specific systemic cytoprotective responses. **a** Representative epifluorescent microscopic images of DAF-16::GFP nuclear translocation in response to a 50-min 35 °C heat stress (HS) or a 30-min exposure to 1 μl ccBA or 4 μl ccDA in young adults. **b** Representative epifluorescent microscopic images of SKN-1::GFP nuclear translocation in response to 4 mM paraquat (PQ) for 1 h, 1 μl ccBA or 4 μl ccDA for 30 min in L3 larvae. Please note the specific fluorescence of the larger nuclei labeled by arrows in the image and in the inset of some samples and the granular intestinal autofluorescence present in each sample. **c** Representative epifluorescent microscopic images of *cyp-35B1p::gfp* expression in response to a 4-h exposure of 1 μl ccBA or 4 μl ccDA in worms fed by control empty vector (EV) and *daf-16* RNAi. **d** Representative epifluorescent microscopic images of *gst-4::gfp* expression in response to a 4-h exposure of 1 μl ccBA or 4 μl ccDA in nematodes fed by EV and *skn-1* RNAi. **e** Quantification of *cyp-35B1p::gfp* expression in response to a 4-h exposure to ccBA or ccDA in worms fed by control EV (top row of panel **c**). **f** Quantification of *gst-4::gfp* expression in response to a 4-h exposure to ccBA or ccDA in nematodes fed by control EV (top row of panel **d**). Please note the lack of detectable fluorescent signal in nematodes fed by *daf-16* or *skn-1* RNAi. Data are expressed as mean ± SEM. *N*, number of independent experiments; *n*, number of animals. *p* values were obtained by one-way ANOVA with Fisher’s LSD post hoc test (**e**) or unpaired Student’s *t* test following the evaluation of normal distribution significance by the Kolmogorov-Smirnov test (**f**). The inter-experimental variation (%CV) for (**f**) was 20% (control), 22% (ccBA), and 25% (ccDA). n.s., not significant; ***p < 0.001

Next, we asked if the efficient vs. insufficient physiological protection against ccBA and ccDA exposure might be reflected in the differential mobilization of cellular defense responses to the respective toxic stresses. In agreement with our findings on the toxicity of ccBA, previous studies demonstrated that BA induced oxidative stress [26]. Therefore, we tested various oxidative stress response pathways that might be involved in the physiological adaptation to ccBA. Using the TJ356 strain expressing GFP-tagged DAF-16, we observed that the same ccBA dose used for preconditioning induced a strong nuclear translocation of DAF-16 after 30 min, comparable to that induced by heat stress. However, DAF-16 remained cytosolic in response to ccDA (Fig. 3a and Additional File 1: Fig. S3a). The shift in DAF-16 localization exhibited a clear BA dose dependence (Additional File 1: Fig. S3b). These congruent ccBA dose-dependent changes in DAF-16 translocation and food avoidance (cf. Fig. 1b and Additional File 1: Fig. S3b) may represent a potential link between cytoprotective responses and behavioral tolerance.

Next, we tested several other stress and detoxification pathways using GFP-tagged marker strains. Translocation of the oxidative-xenobiotic stress master regulator SKN-1::GFP in the LD001 strain was induced by a 30-min exposure to ccBA, comparable to that seen upon the oxidative agent paraquat (PQ) treatment, but not by ccDA (Fig. 3b). Further, ccBA, but not ccDA, induced the expression of xenobiotic-metabolizing reporters: the phase I oxidative cytochrome P450 enzyme *cyp-35B1* and the phase II conjugating enzyme *gst-4* (Fig. 3c–f) involved in the detoxification of lipophilic compounds. The induction of *cyp-35B1* was abolished by *daf-16* RNAi, while that of *gst-4* was abolished by *skn-1* RNAi (Fig. 3c, d). Importantly, ccBA did not activate several other stress reporters, including the HSF-1 and DAF-16 target *hsp-16.2*, the HSF-1 target and endoplasmic reticulum unfolded protein response (UPR) reporter *hsp-4*, the SKN-1-dependent *gcs-1*, and the DAF-16-dependent *sod-3* reporter (Additional File 1: Fig. S3c). These findings demonstrate that a specific stress and detoxification response involving a subset of DAF-16- and SKN-1-activated genes participate in the molecular defense against ccBA toxicity. In contrast, no apparent stress responses were detected upon ccDA exposure.

### ccBA-induced cytoprotective responses confer behavioral tolerance to ccBA, but not to ccDA

**Fig. 4:**
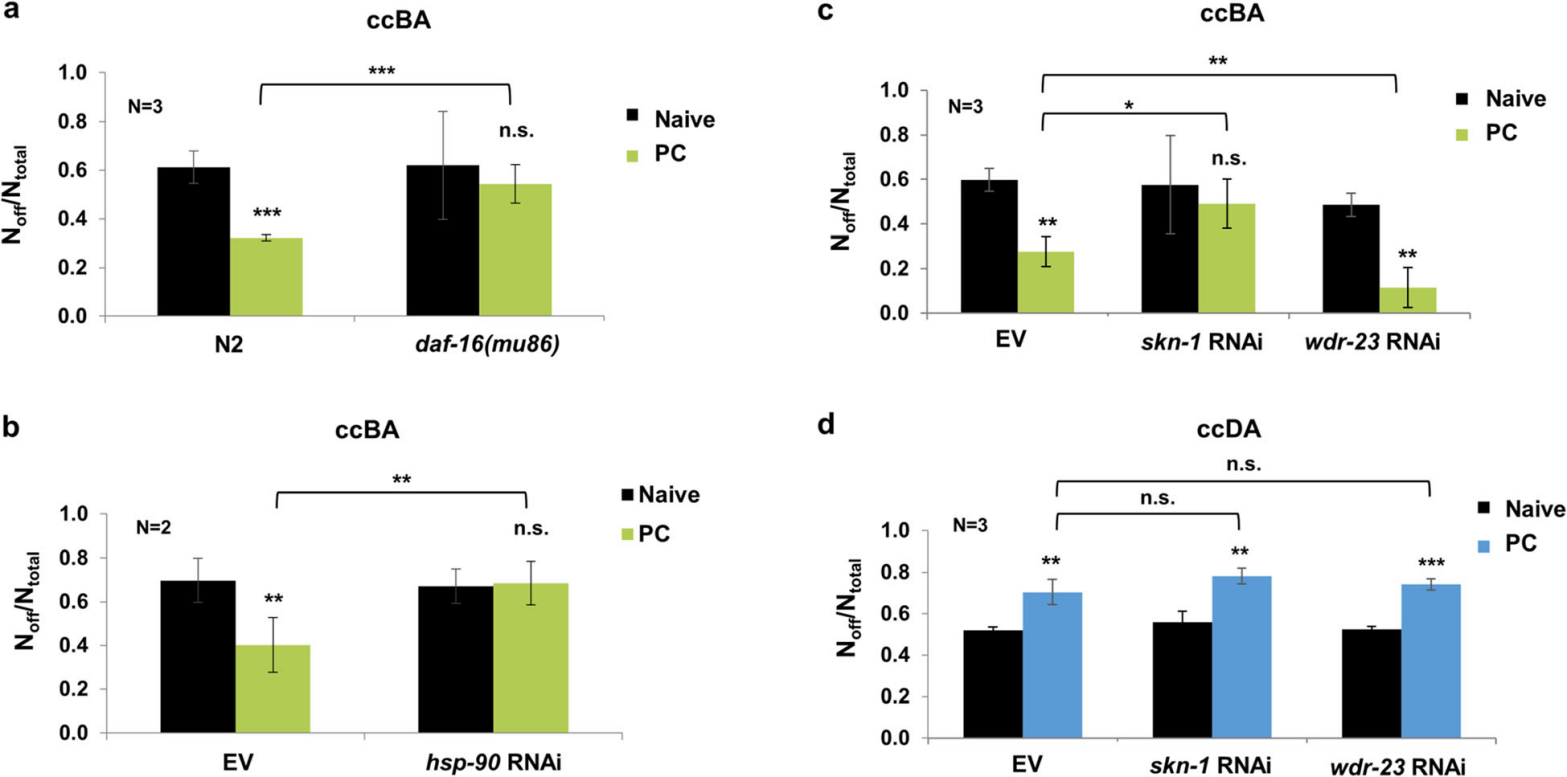
ccBA-induced cytoprotective responses in non-neuronal cells confer behavioral tolerance to ccBA, but not to ccDA. **a** ccBA-induced food aversion of naive and ccBA-preconditioned (PC) N2 wild-type and *daf-16(mu86)* mutant animals. **b** ccBA-induced food aversion of naive and ccBA-preconditioned nematodes fed by control empty vector (EV) and *hsp-90* RNAi bacteria. **c** ccBA-induced food aversion of naive and ccBA-preconditioned nematodes fed by EV, *skn-1*, and *wdr-23* RNAi, respectively. **d** ccDA-induced food aversion of naive and ccDA preconditioned nematodes fed by control EV, *skn-1*, and *wdr-23* RNAi, respectively. Preconditioning and food leaving experiments were performed as indicated in Fig. 2. Data are expressed as mean ± SEM. *N*, number of independent experiments. *p* values were obtained by one-way ANOVA with Fisher’s LSD post hoc test. n.s., not significant; *p < 0.05; **p < 0.01; ***p < 0.001

The “fight-or-flight” response is an essential part of the general adaptation reaction to diverse stresses [2]. Therefore, we asked whether the cytoprotective responses activated by ccBA which are known to induce physiological tolerance to various stresses might play a role in the generation of “fight-or-flight” (staying on or leaving the lawn) behavioral decisions. To this end, we preconditioned N2 and *daf-16* null mutant nematodes with ccBA and studied their food avoidance to ccBA. We found that naive *daf-16* mutants showed avoidant behavior comparable to wild type; however, they failed to decrease their aversion in response to preconditioning (Fig. 4a). A similar phenotype was obtained by silencing the evolutionarily conserved molecular chaperone Hsp90, which was shown to regulate DAF-16 activity [27] (Fig. 4b). Likewise, *skn-1* silencing similarly prevented the development of behavioral tolerance, whereas the activation of SKN-1 by knocking down the WDR-23 protein responsible for its degradation [28] augmented behavioral tolerance towards ccBA (Fig. 4c). Further, RNAi knockdown of *daf-16* or *skn-1* impaired survival to ccBA (Additional File 1: Fig. S4). In sharp contrast, after ccDA preconditioning, neither *skn-1* nor *wdr-23* RNAi altered the behavioral sensitization towards ccDA exposure (Fig. 4d). RNAi did not silence neuronal Hsp90 and SKN-1 isoforms [16, 27], in agreement with its inability to enter neurons [29]. These results demonstrate that specific cytoprotective responses induced by toxic ccBA exposure in non-neuronal cells confer physiological protection and actively participate in the development of behavioral tolerance. Thus, the ability to mount stress-specific molecular “fight” responses downregulates the behavioral avoidance “flight” response.

### Behavioral cross-tolerance is mediated by chemical structure-specific cytoprotective responses

**Fig. 5:**
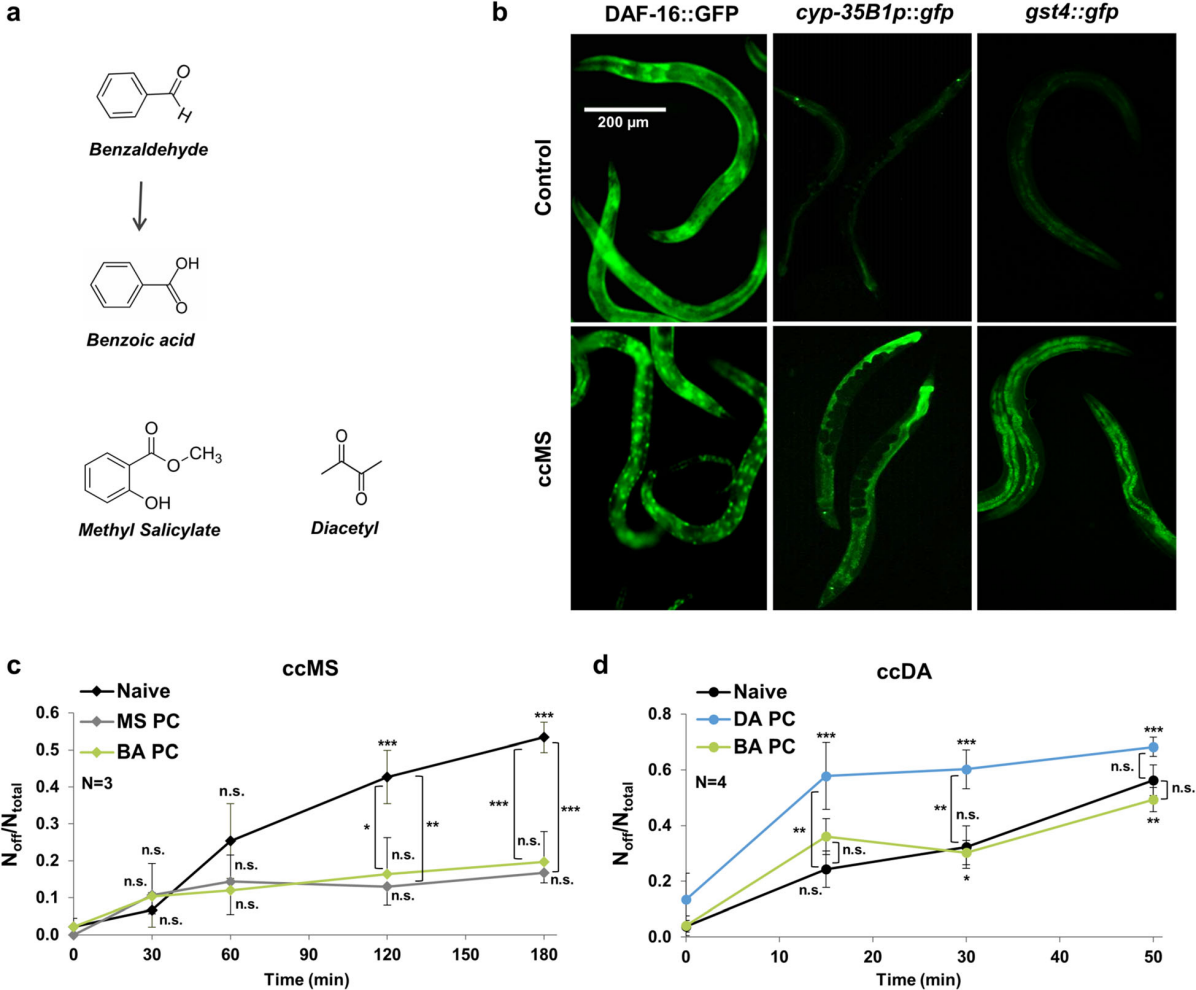
Behavioral cross-tolerance is mediated by chemical structure-specific cytoprotective responses. **a** Benzaldehyde, its metabolite benzoic acid, and methyl-salicylate (MS) share similar chemical structures. **b** Representative epifluorescent microscopic images showing the effect of a 30-min exposure of 1 μl undiluted MS (ccMS) on DAF-16::GFP nuclear translocation and a 4-h of 1 μl ccMS exposure on *cyp-35B1p*::*gfp* and *gst-4::gfp* reporters expression. **c** Effect of preconditioning with ccMS (1 μl for 4 h, MS PC) or ccBA (BA PC) on 1 μl ccMS-induced lawn avoidance. **d** Effect of preconditioning with ccDA (DA PC) or ccBA on ccDA-induced lawn avoidance. Preconditioning and food leaving experiments were performed as indicated in Fig. 2. Error bars represent mean ± SEM compared to the respective naive values. *N*, number of independent experiments. *p* values were obtained by one-way ANOVA with Fisher’s LSD post hoc test. n.s., not significant; **p < 0.01, ***p < 0.001

Xenobiotic-induced stress and detoxification responses are specific to the chemical structure of the toxin and to the nature of damage they induce [30]. We reasoned that the observed BA-dependent cytoprotective machinery might also be induced in response to a chemically similar toxic compound. BA is both spontaneously and enzymatically oxidized to benzoic acid during its detoxification [26, 31] (Fig. 5a). The chemical structure of the volatile plant stress hormone methyl-salicylate (MS) [32], harboring an aromatic benzene ring and an esterified carboxyl group, is closely related to that of benzaldehyde and benzoic acid (Fig. 5a). We found that similarly to ccBA, undiluted MS (ccMS) was also toxic and induced food avoidance behavior (Additional File 1: Fig. S5a-c). Moreover, ccMS and ccBA shared identical, sustained molecular defense responses, including DAF-16 translocation, *cyp-35B1p::gfp*, and *gst-4::gfp* expression (Fig. 5b). Importantly, preconditioning with either ccMS or ccBA reduced food aversion in response to a subsequent ccMS exposure (Fig. 5c). However, ccBA preconditioning did not affect food aversion in the presence of ccDA, indicating that ccBA-activated including DAF-16- and SKN-1-dependent processes are unable to reduce ccDA toxicity (Fig. 5d). We conclude that the BA-specific cytoprotective responses confer behavioral cross-tolerance towards a toxin harboring a similar chemical structure, but not towards another compound, DA, which is unrelated chemically and probably by the mechanism of action.

### JNK-like MAP kinases and the NPR-1 neuropeptide Y receptor connect behavioral and physiological stress tolerance

**Fig. 6:**
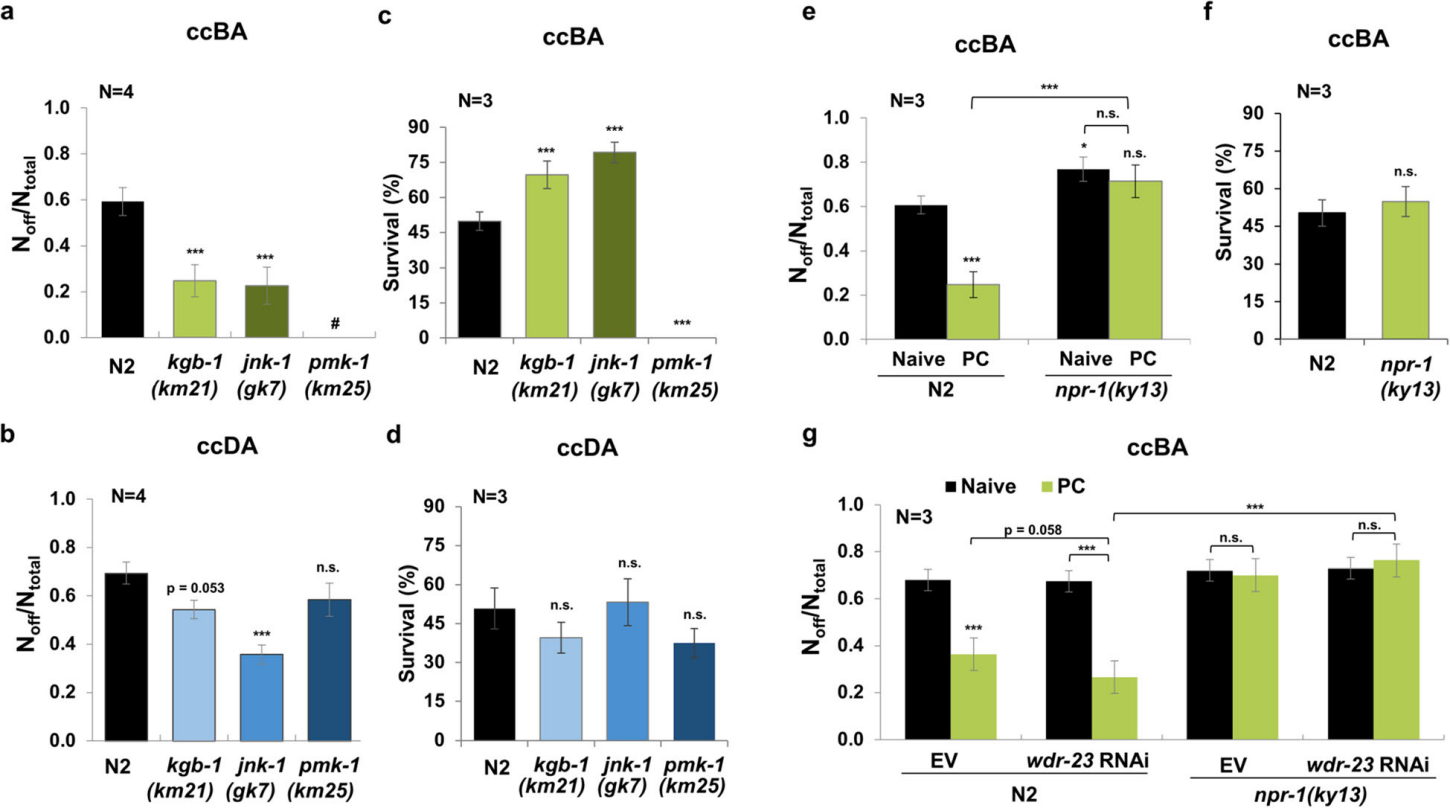
JNK-like MAP kinases and NPR-1 connect behavioral and physiological stress tolerance. **a** ccBA-induced food aversion of wild-type and SAPK mutant worms. **b** ccDA-induced food aversion of wild-type and SAPK mutant worms. **c** Survival of wild-type and SAPK mutant worms 14 h after a 3-h exposure to 8 μl ccBA. **d** Survival of wild-type and SAPK mutant worms 14 h after 3-h exposure to 16 μl ccDA. **e** ccBA-induced food aversion of naive and ccBA-preconditioned (1 μl for 4 h) N2 and *npr-1* mutants. **f** Survival of N2 and *npr-1* mutants 14 h after exposure to 8 μl ccBA for 3 h. **g** ccBA-induced food aversion of naive and ccBA-preconditioned (1 μl for 4 h) N2 and *npr-1* mutants, fed by control empty vector (EV) or *wdr-23* RNAi. Preconditioning and food leaving experiments were performed as indicated in Fig. 2. Data are expressed as mean ± SEM. *N*, number of independent experiments. *p* values were obtained by one-way ANOVA with Fisher’s LSD post hoc test. n.s., not significant; ***p < 0.001

The effect of extraneuronal and intracellular defenses in behavioral modulation upon stress suggested the involvement of inter-tissue signaling mechanisms. In eukaryotes, the conserved stress-activated protein kinase (SAPK) signaling pathways are activated in adversity and facilitate protective organismal responses in a coordinated manner [33]. In *C. elegans*, the major downstream MAP kinases including the p38 ortholog PMK-1 as well as the JNK orthologs JNK-1 and KGB-1 guard physiological homeostasis in diverse stresses [34]. Besides, a requirement of *kgb-1* in avoidance of toxic lawns [18] and inhibition of the *Pseudomonas aeruginosa* pathogen avoidance by *pmk-1* have been reported [35]. Hence, we tested the involvement of the respective mutants in ccBA aversion by subjecting naive and ccBA-preconditioned worms to the ccBA lawn leaving assay. Both *kgb-1* and *jnk-1* mutations diminished the aversion of naive worms to levels reminiscent of ccBA-preconditioned wild type (Fig. 6a), whereas loss-of-function of *sgk-1*, an unrelated kinase was without significant effect (Additional File 1: Fig. S6a). *pmk-1* mutants rapidly and irreversibly paralyzed and died on the otherwise non-paralytic dose of ccBA; therefore, its role in ccBA avoidance could not be evaluated (Fig. 6a, Additional File 1: S6b). Avoidance to ccDA also required, though a smaller extent, *jnk-1* and perhaps *kgb-1*, which was at the threshold of significance, whereas *pmk-1* exerted no significant effect (Fig. 6b). These results suggest a role for JNK-like kinases in toxic odorant-elicited aversive behavior.

SAPK members exert specific and overlapping roles in physiological defenses against various stresses. All three kinases help combat proteotoxic and heavy metal stress [34]. Besides, PMK-1 promotes oxidative/xenobiotic, osmotic, and pathogen resistance partly via SKN-1 [15, 16, 36]. JNK-1 promotes heat stress resistance via DAF-16 [37], while KGB-1 is required to protect from bacterial pore-forming toxins and ER stress [38, 39]. Hence, a parallel stimulation of behavioral aversion and physiological defenses by JNK-like kinases might be feasible. Therefore, we exposed SAPK mutants to the lethal dose of the respective odors and tested their survival. Contrary to our assumption, *kgb-1* and *jnk-1* mutants, compared to wild-type, exhibited enhanced survival upon ccBA and unchanged survival upon ccDA exposure (Fig. 6c, d). These results are consistent with the lack of specific physiological defenses against ccDA, and a reciprocal effect of JNK-like kinases on ccBA-elicited responses: promotion of behavioral avoidance and attenuation of ccBA-specific physiological defenses (cf. Fig. 6a–d). As the ccBA concentration in the survival plates is uniform, the increased survival of *kgb-1* and *jnk-1* is independent of their reduced aversion. Therefore, either the JNK-like kinases separately promote aversion and suppress physiological stress responses or the suppression of stress responses indirectly promotes aversion. Although our results do not allow a clear distinction, both alternatives confirm the reciprocal connection between physiological and behavioral defenses, observed with the cytoprotective regulators. Loss of *pmk-1* function did not significantly affect survival on ccDA (Fig. 6d), but completely hindered survival on ccBA (Fig. 6c), in agreement with the extensive paralysis observed on low-dose ccBA. Altogether, these findings suggest a physiological protection of vital importance conferred by *pmk-1* against ccBA toxicity, a requirement of JNK-like kinases to favor behavioral defense vs. ccBA-specific physiological defenses, and *jnk-*1 (and *kgb-1*) to elicit avoidance as the sole available protective measure against ccDA.

The conserved neuropeptide Y receptor ortholog NPR-1 is an important integrator of various external and internal cues and modulates diverse physiological and behavioral responses including innate immunity, social vs. solitary feeding, arousal, and avoidance of *P. aeruginosa* [40–42]. We investigated the behavioral response of naive and ccBA-preconditioned *npr-1* mutants to ccBA in food leaving assays. *npr-1* mutants initially aggregated on the *E: coli* lawn, but in response to ccBA, they dispersed and left the lawn, similarly to wild-type animals. Strikingly, we observed a complete suppression of the behavioral tolerance in ccBA-preconditioned *npr-1* mutants (Fig. 6e). The increased aversive behavior of *npr-*1 mutants could ensue from a compromised resistance to ccBA toxicity, as NPR-1 activates physiological defenses, such as PMK-1-dependent immunity in response to *P. aeruginosa* infection [41]. However, the *npr-1* mutation did not affect survival upon lethal ccBA exposure (Fig. 6f), suggesting that wild-type NPR-1 does not engage physiological defenses, rather appears to integrate the internal signals of physiological homeostasis into the aversive response against ccBA. We tested this prediction by boosting SKN-1 activity in N2 and *npr-1* animals using *wdr-23* RNAi and subjecting them to the ccBA food leaving assay. Indeed, *wdr-23* RNAi improved the behavioral tolerance in preconditioned wild type, but not in *npr-1* nematodes (Fig. 6g), indicating the disconnection in physiological and behavioral defenses in the absence of *npr-1*. Due to the strong escape of ccBA-preconditoned *npr-1* worms, which phenocopied the avoidance of ccDA lawns by ccDA-preconditioned wild type and the lack of ccDA-induced physiological defenses, we did not study *npr-1* in ccDA conditions. Altogether, these results suggest that SAPK-s and NPR-1 exert opposite effects and cooperate in fine-tuning physiological and behavioral “fight-or-flight” responses to protect homeostasis in toxic stress conditions.

### Deficient or efficient physiological defenses generate relevant learned behaviors to stress-associated olfactory cues

**Fig. 7:**
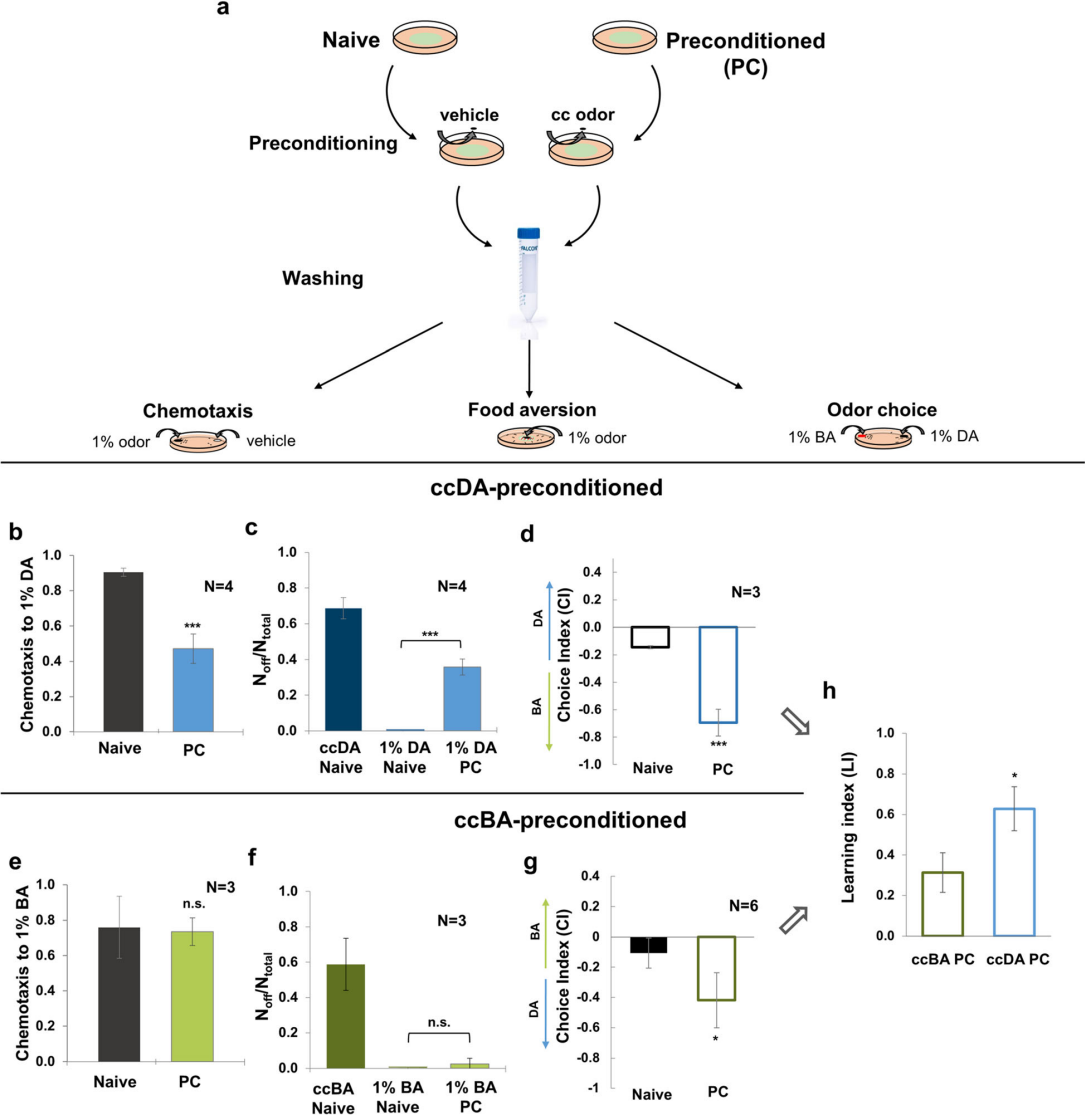
Avoidant and tolerant learned behaviors elicited by stress-associated olfactory cues. **a** Experimental design for the toxic odor preconditioning-induced learning paradigm. Animals were exposed to a hanging drop of undiluted odor (preconditioned, PC) or vehicle (naive), washed, and assayed for chemotaxis, food aversion, and odor preference using diluted, 1% odors. **b** Effect of ccDA preconditioning on chemotaxis to 1% DA. **c** Effect of ccDA preconditioning on lawn avoidance in the presence of 1% DA. **d** Effect of ccDA preconditioning on odor choice between 1% DA and 1% BA. Choice indices were calculated as CI = (# on DA − # on BA)/(# on DA + # on BA). **e** The effect of ccBA preconditioning on chemotaxis to 1% BA. **f** Effect of ccBA preconditioning on lawn avoidance in the presence of 1% BA. **g** Effect of ccBA preconditioning on odor choice between 1% BA and 1% DA. Choice indices were calculated as CI = (# on BA − # on DA)/(# on BA + # on DA). **h** Learning indices from **e** and i, calculated as LI = CI (naive) − CI (preconditoned). Error bars represent mean ± SEM. *N*, number of independent experiments. *p* values were obtained by one-way ANOVA (for chemotaxis and food leaving assays) and by two-way ANOVA (for odor choice assays) with Fisher’s LSD post hoc test. n.s., not significant; *p < 0.05; **p < 0.01; ***p < 0.001

After an initial attraction to pathogenic or toxin-producing bacteria, *C. elegans* develops behavioral aversion through a process called avoidance learning, which is driven by neural associations between the internal experience of stress and the co-occurring chemosensory cues [18, 23, 42]. We asked whether the prior experience of odor toxicity and the different efficiency of physiological defenses influence nematodes to make optimal choices upon encounters with the olfactory cues present at the time of stress. To examine this, we investigated alterations in behaviors towards attractive (1%) doses of DA and BA after preconditioning with toxic, undiluted doses of the respective odors (Fig. 7a). We speculated that the history of uncompensated stress induced by ccDA might predispose animals to avoid the naturally attractive DA cue. As expected, we found that worms preconditioned with ccDA significantly reduced their chemotaxis towards 1% DA by 50% (Fig. 7b). Importantly, almost 40% of the animals chose to leave the food in the presence of 1% DA after ccDA preconditioning, corresponding to over half of the worms that avoided the lawn containing ccDA (Fig. 7c). We also examined how stress history affects the decision between DA and another attractive food olfactory cue by providing both DA and BA in an odor choice assay. The aversive change of the DA olfactory cue was underscored by an almost complete shift in odor preference to BA (Fig. 7d). It is plausible that adaptation towards DA may contribute to the decreased chemotaxis and odor preference. Adaptation to olfactory cues might occur under starvation, but the presence of food during preconditioning in our experiments prevents habituation and largely suppresses adaptation [24]. However, even if worms might adapt to DA, the high lawn avoidance and the comparability to the decrease in chemotaxis suggest that the observed behavioral changes are predominantly caused by learned avoidance of the anticipated threat.

In contrast, worms preconditioned with ccBA maintained their chemotaxis towards 1% BA when the olfactory cue of BA was the only option (Fig. 7e). This result shows that preconditioning did not cause adaptation to BA. Moreover, they did not leave the bacterial lawn in the presence of 1% BA (Fig. 7f). Remarkably, worms displayed reduced preference to BA in the simultaneous presence of DA (Fig. 7g). Taken together, worms exhibit selective avoidance in response to the olfactory cue they encountered during toxic stress, representing the development of learned avoidance. In agreement with this, the positive learning indices indicate that odorant-preconditioned worms learn to avoid both odor cues with a stress history (Fig. 7h). However, the smaller learning index as well as the preserved chemotaxis and lawn occupancy in case of BA suggests a flexibility in learned behavioral decisions. These results are consistent with the formation of distinct, avoidant, or tolerant learned behaviors, respectively, which appear to stem from the prior internal experience of deficient or efficient cytoprotection.

### A memory of physiological stress resistance enables flexible decision-making

**Fig. 8:**
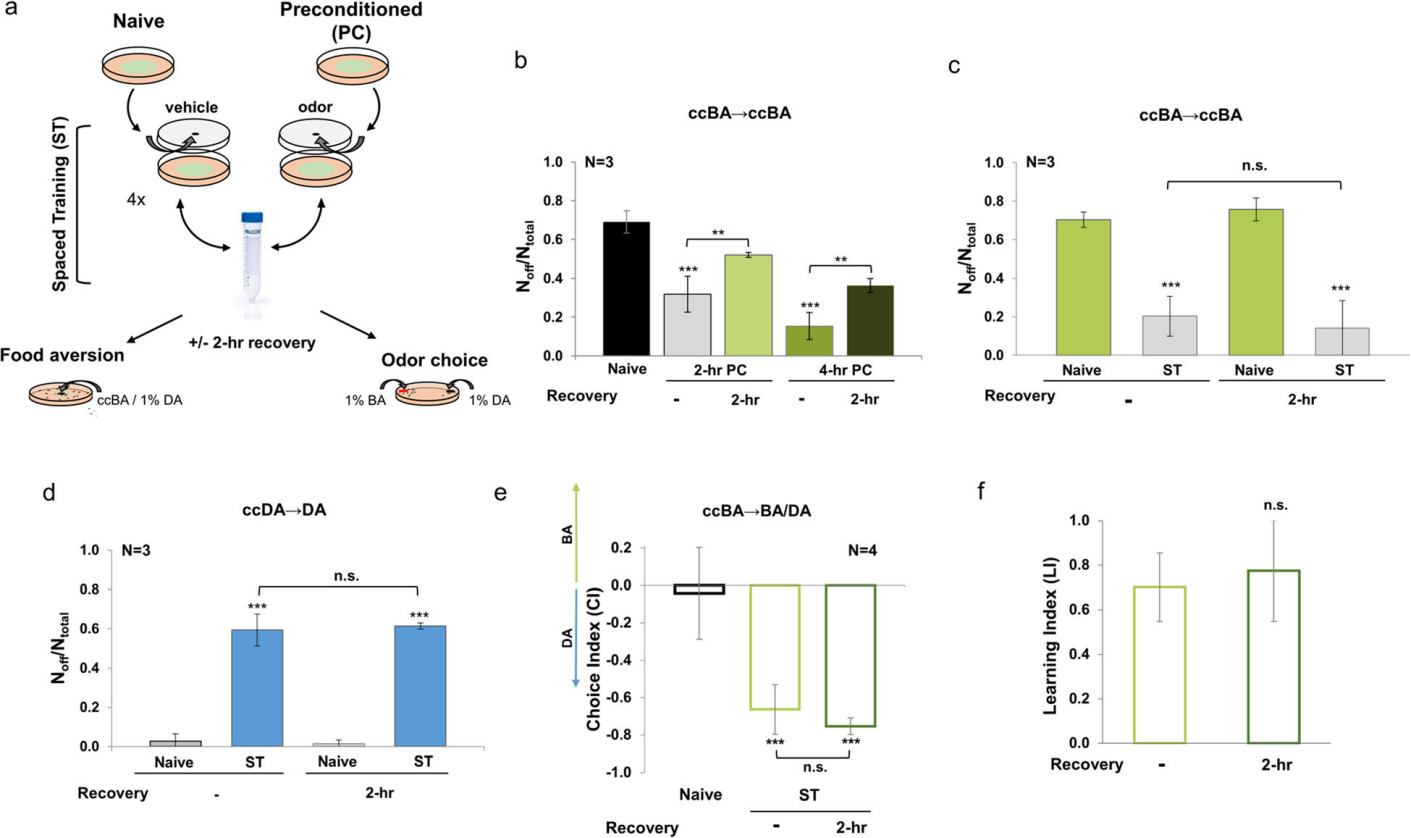
Reinforcement of stress-associated experiences forms distinctive avoidant and coping memories. **a** Experimental design for spaced training (ST) using toxic odors. Animals were exposed to a hanging drop of undiluted odor (2 μl ccBA or 4 μl ccDA, preconditioned, PC) or vehicle (naive) using a 4 × 1-h spaced training protocol with 10-min inter-trial “rest” times during the washing step. Animals were assayed for food aversion or odor preference immediately or after a 2-h recovery period. **b** Effect of a 2-h recovery period on ccBA-induced food aversion elicited by ccBA preconditioning using single 2-h (2-h PC) or 4-h (4-h PC) exposures. **c** Effect of a 2-h recovery period on ccBA-induced food aversion elicited by ccBA spaced training. **d** Effect of a 2-h recovery period on lawn avoidance in the presence of 1% DA elicited by ccDA spaced training. **e** Effect of a 2-h recovery period on odor choice between 1% BA and DA elicited by ccBA spaced training. Choice indices were calculated as CI = (# on BA − # on DA)/(# on BA + # on DA). **f** Learning indices from **e**, calculated as LI = CI (naive) − CI (preconditioned). Error bars represent mean ± SEM. *N*, number of independent experiments. *p* values were obtained by one-way ANOVA (for food leaving assays) and by two-way ANOVA (for odor choice assays) with Fisher’s LSD post hoc test.t. n.s., not significant; **p < 0.01, ***p < 0.001

The elicitation of learned stress-reactive behaviors by the respective olfactory cues raises the possibility that the learned experiences may give rise to distinct memories to cope with anticipated future insults. On the other hand, forgetting irrelevant, non-recurrent experiences is also important as both the organism and the environment are changing. We tested the stability of newly acquired behaviors by subjecting worms to ccBA lawn leaving assays immediately or 2 h after preconditioning with ccBA. We observed that a 2-h recovery period after a single ccBA preconditioning for 2 or 4 h significantly attenuated behavioral tolerance against ccBA in the food leaving assay (Fig. 8b). The increased lawn avoidance after recovery might either be due to the decrease in stress-induced physiological defenses or in the loss of the new, yet unstable changes in neural representation, forgetting. Repeated training sessions with inter-trial “rest” intervals, called spaced training, potently amplifies learning efficiency via memory consolidation [43]. Spaced training is known to induce stable memories over 2 h in *C. elegans* [44]. Hence, we tested whether spaced training, by counteracting forgetting, might increase the persistence of the acquired behavioral tolerance to ccBA after the recovery. The induction of aversive memory is optimal beyond three training sessions with 10-min “rest” intervals [45]. Therefore, we employed a protocol of spaced training using four times 1-h exposures to 2 μl ccBA on large food lawns with 10-min rest times during the washing steps in between (Fig. 8a). Doubling the ccBA dose compared to the single 4-h preconditioning protocol was to compensate for ccBA-free rest periods. Then, half of the nematodes were subjected to ccBA lawn leaving assays immediately after training, the other half after a 2-h recovery period. We found that immediately after pre-exposures, both the single preconditioning and the spaced training resulted in a similar suppression of ccBA avoidance, suggesting similar levels of behavioral tolerance elicited by both protocols (Fig. 8b, c). However, the behavioral tolerance was entirely retained after a 2-h recovery in spaced-trained nematodes (Fig. 8c). We also examined whether repetitive encounters with the same dose of ccDA as in single preconditioning might influence food avoidance behavior in the presence of 1% DA. We observed that spaced-trained worms exhibited robustly increased food leaving behavior against 1% DA (Fig. 8d), compared to that elicited by a single 4-h preconditioning (see Fig. 6c, “DA PC” column), reaching a similar aversion index to that elicited by ccDA (see Fig. 6c, “ccDA Naive” column). Moreover, the extent of the avoidant behavior was entirely preserved after the 2-h recovery (Fig. 7d). Thus, spaced training with ccBA or ccDA leads to the stabilization of respective stress-associated memories over 2 h, which upon retrieval give rise to either tolerant, coping “fight,” or avoidant “flight” behavioral responses.

Finally, we asked how the coping memory affects the choice between the stress-associated and a natural attractive odor olfactory cue. Spaced training with ccBA almost entirely shifted the preference towards DA (Fig. 8e), potentiating the change already observed by the single preconditioning (see Fig. 7g, h). Moreover, the robustly shifted odor preference evoked by spaced training was retained after a 2-h recovery (Fig. 8e) resulting in stable storage and retrieval of the acquired memory (Fig. 8f). The stability of memories generated by spaced training is consistent with the literature data [44, 45]. Hence, reinforcement of learning by spaced training led to the augmentation and stabilization of the acquired behavioral changes induced by ccBA and ccDA. The complete shift of preference from BA to DA shows an apparent similarity to the complete shift of preference from DA to BA after the single preconditioning with ccDA (cf. Figs. 8e, f and 7d, h). Nonetheless, in contrast to the compelling avoidant “flight” behavior to the memory of uncompensated physiological harm, the memory of physiological protection not only provides the ability to cope with real or anticipated toxicity for food, but also allows a flexible decision to spare resources when the organism also perceives the olfactory cue of a potentially toxin-free food. This result also suggests that the memory of a stressful insult contains the representation of the original valence of the olfactory cue, the internal experience of stress-induced harm, and the activated physiological protection.

## Discussion

**Fig. 9:**
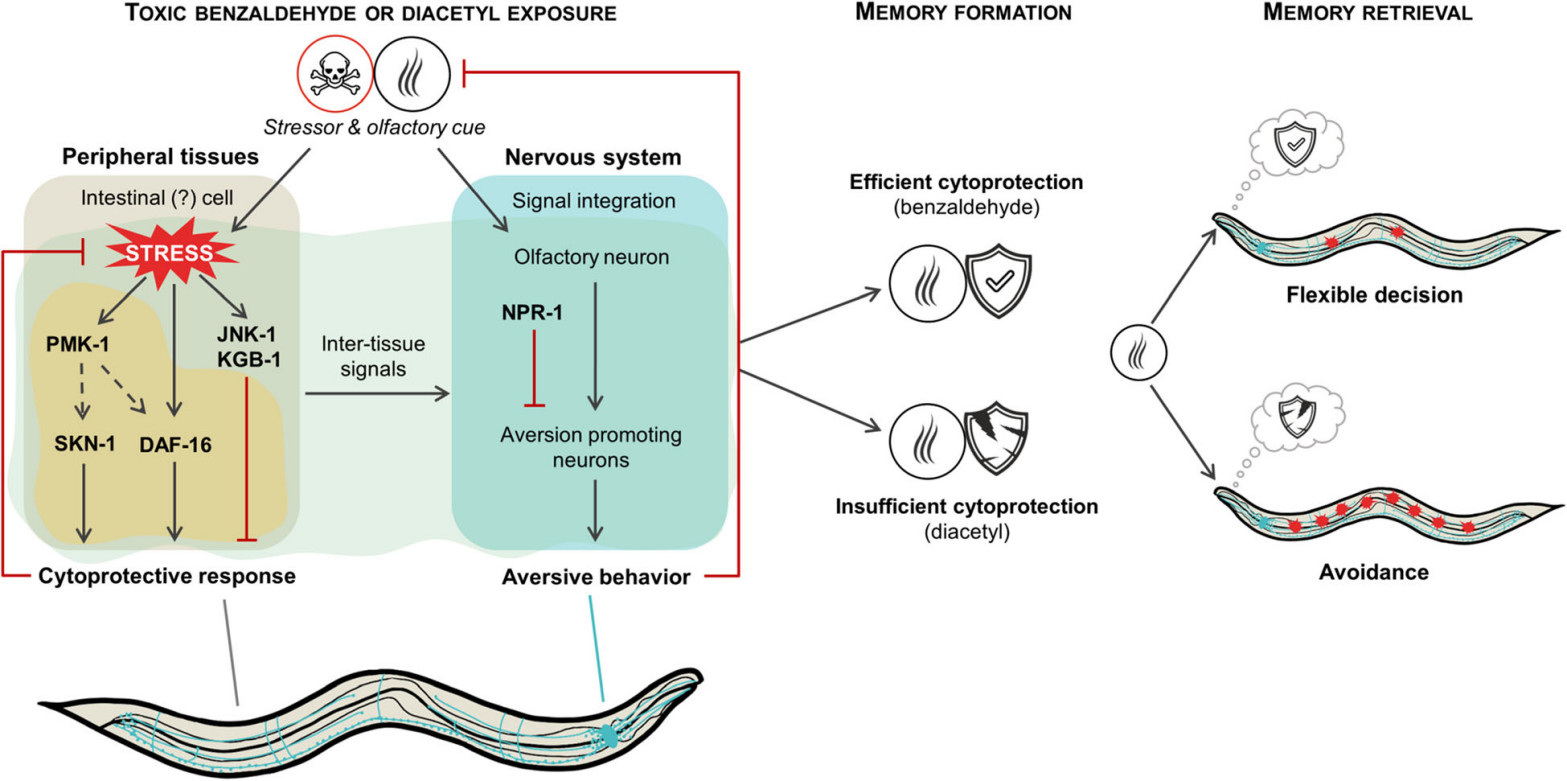
Model for the regulation of learned behavioral decisions by cytoprotective responses. Undiluted odorants induce stress in non-neuronal cells. Cells emit inter-tissue danger signals to the nervous system which require JNK-like kinases and are integrated with other signals to control aversion. (The site of action of KGB-1 and JNK-1, although indicated in the peripheral cell, is yet undefined.) Benzaldehyde-specific cytoprotective responses (beige area) alleviate stress and danger signals, which diminish aversion *via* the neuropeptide receptor NPR-1. Reinforcement of these experiences forms a memory of protection, which upon retrieval by the olfactory cue allows a flexible decision depending on the external context, such as the availability of other, stress-free food sources. Insufficient cytoprotection (diacetyl) aggravates stress which leads to behavioral sensitization and forms a memory of danger, which upon retrieval compels to avoidance. Dashed lines denote results inferred from other studies, see the “Discussion” section for details

In this study, we have set up a toxic stress paradigm in *C. elegans* to assess the impact of cytoprotective responses on behavioral decisions. We have shown that the innately attractive odorants benzaldehyde and diacetyl, when employed at high concentrations, induce toxicity and behavioral aversion of the odorant-contaminated bacterial lawn. ccBA preconditioning-induced cytoprotective responses in non-neuronal cells involving DAF-16, SKN-1, and Hsp90 conferred physiological and behavioral tolerance to ccBA and cross-tolerance to undiluted methyl-salicylate (ccMS), while neither behavioral tolerance nor apparent physiological defenses were observed upon exposure to ccDA. We found that the connection between cellular stress defense and behavioral avoidance requires inter-tissue signals relayed by JNK-like stress-activated MAP kinases and the neuropeptide Y receptor NPR-1. Spaced training generated a memory that made diluted DA aversive but enabled animals to decide whether to approach or to avoid BA depending on alternative choice. Our study suggests that the (in)ability of *C. elegans*’ cells to counteract toxic stress with cytoprotective mechanisms regulates behavior during stress and determines learned behavioral decisions upon re-encounter with stress-associated olfactory cues (Fig. 9).

Our observations on the toxic odorant-induced aversive behavior confirm earlier studies that showed aversive properties of high concentrations of food-derived attractive volatiles in the absence of food [10, 21, 22]. The exposure to the undiluted odorants on bacterial lawns and monitoring the lawn leaving behavior eliminated the habituation or olfactory adaptation phenomena arising when the odor is the sole chemosensory stimulus. The association with organismal toxicity, the phenomenon of behavioral cross-tolerance to a different odorant, the involvement of conserved cytoprotective responses in non-neuronal cells, and the inter-tissue regulatory signals together show that aversion is a systemic behavioral defense against toxic stress. Consistent with our study, various studies in mammals describe the toxic effects of benzaldehyde [26] and diacetyl [46]. The conservation of physiological responses, the toxicity, and the toxic stress model presented here suggest *C. elegans* as a suitable model to study the toxic effects of volatiles. Our findings also draw attention to nematode associative learning experiments where different conditions are paired with undiluted odorants, because odorant toxicity might stimulate repulsion independently of, or synergistically with, the unconditioned stimulus. Therefore, behavioral experiments using diluted odorants as the conditioned stimulus might be considered.

Results of the food leaving experiments also show that the behavioral avoidance “flight” response is the first line of defense against dangerous insults, which preserves physical health and spares resources. ccBA-exposed worms, however, started to return to food during the second hour, and a preconditioning exposure also diminished ccBA avoidance. Reduced avoidance coincided with DAF-16 and SKN-1 activation and induction of phase 1 and 2 xenobiotic detoxification reporters, consistent with the aromatic structure and toxic profile of ccBA. Such cytoprotective stress and detoxification responses form a cellular defense network and cooperate to ensure survival, stress tolerance, immunity, and longevity [3, 12]. A few studies reported on the neural “top-down” control of cellular cytoprotective responses in worms [20, 41, 47], but a “bottom-up” direction of communication is much less explored. Such regulation has only recently been observed in the case of the inhibition of *P. aeruginosa* avoidance by intestinal *pmk-1* [35]. Likewise, our findings that *daf-16* knockout, *hsp-90*, *skn-1*, and *wdr-23* RNAi in non-neuronal cells specifically modulate ccBA avoidance show that stress-specific cytoprotective regulators control aversion. The apparent impermeability of the cuticle to chemicals [48], the localization of DAF-16- and SKN-1-positive nuclei around the gut lumen, and the predominant localization of SKN-1 and DAF-16 isoforms in the intestine suggest a key role of the gut in the regulation of behavioral defenses, which is consistent with its roles as the major inner barrier and site of immunity and detoxification. Altogether, our findings reveal a novel regulatory role of peripheral cytoprotective stress responses on behavioral decisions.

We found ccBA-induced behavioral cross-tolerance to undiluted methyl-salicylate (ccMS). Our results on identical cytoprotective responses shared by ccBA and ccMS suggest that the responses stimulated by ccBA preconditioning also protect tissue homeostasis during ccMS exposure. Indeed, high doses of methyl-salicylate cause heavy toxicity in mammals [49]. We propose that the preservation of tissue homeostasis by toxin- and damage-specific cytoprotective responses suppresses aversion. Consistent with this idea, ccDA preconditioning highly accelerated ccDA aversion and robustly increased lethality upon a subsequent ccDA exposure. Furthermore, ccDA did not appear to activate considerable molecular defenses, and neither SKN-1 manipulations nor the systematic induction of cellular defenses by ccBA preconditioning affected ccDA aversion. These results also exclude the unlikely possibility that induction of systemic cytoprotective responses *per se* inhibits aversive behavior. Hence, the disturbance of cellular homeostasis may lead to the emission of yet unknown danger signals, which upon detection will execute the neuronal aversive response.

The modulation of aversion by non-neuronal RNAi manipulations suggests an inter-tissue signal transmission mechanism. The stress-activated JNK and p38 MAP kinases are conserved signal transducers of cell stress and orchestrators of physiological cytoprotective responses against xenobiotic, oxidative, proteotoxic, genotoxic, and pathogen stresses [33]. Previous studies in *C. elegans* showed that the JNK-1 ortholog KGB-1 mediates [18], whereas the p38 ortholog PMK-1 [35] inhibits, aversion in response to toxicity and infection, respectively. Our results obtained with SAPK mutants show no role in aversion for *pmk-1* and confirm the requirement of *kgb-1* for aversion against ccBA and a marginal role against ccDA. Remarkably, the results obtained against both ccBA and ccDA reveal a hitherto unidentified requirement of *jnk-1* in behavioral aversion. The modest requirement of the JNK-like kinases in ccDA avoidance suggests either a redundant action or other, yet unknown players in the behavioral response against ccDA toxicity. It is interesting to note that loss of JNK-like kinases inhibited ccBA aversion to levels mimicking wild-type behavioral tolerance (compare Fig. 6a naive *kgb-1* and *jnk-1* to Fig. 6e N2 PC). The surprising elevation of survival upon ccBA exposure in both *kgb-1* and *jnk-1* mutants sheds light on a reciprocal regulation of physiological and behavioral defenses by JNK-like kinases and a coordinated action with the p38 ortholog PMK-1, which was crucial for survival. A major downstream mediator of PMK-1 action is SKN-1 [15, 16]. However, the dramatic effect of *pmk-1* mutation compared to that of *skn-1* RNAi on survival upon ccBA argues for additional, SKN-1-independent routes in PMK-1-orchestrated physiological defenses. The lack of influence of SAPK mutants on survival upon ccDA might reflect the absence of downstream stress responses to confer physiological protection. Our findings do not exclude other regulators of aversion and provide an insight into the cooperative and specific functions stress-activated MAP kinases play in toxic stress defenses, which requires systematic future research.

Contrary to the aversion-promoting effect of JNK-like kinases, we found that NPR-1 is required for the suppression of ccBA avoidance after ccBA preconditioning, in worms with both wild-type and enhanced intestinal SKN-1 activity [16, 28]. This finding suggests a role for NPR-1 in mediating the inhibitory action of cellular defenses on behavioral avoidance. It is of interest that NPR-1 promotes pathogen avoidance in response to intestinal distension [42] and inhibits toxic ccBA-induced avoidance (our study). However, NPR-1 also mediates contrasting immune and behavioral responses against *P. aeruginosa* and *Bacillus thuringiensis* [50], and loss of *npr-1* has also been shown to disrupt locomotor quiescence after cellular damage [51]. These findings suggest that NPR-1 helps integrate various inputs in a context- and stress-dependent manner. Free-living *C. elegans* harbors natural *npr-1* loss-of-function variants [40]; thus, the stress-avoidant phenotype observed in our study may be one significant strategy at the population level. The higher tolerance provided by NPR-1 against the noxious insults, including that of ccBA exposure, may be an evolutionary antecedent of the clinically relevant role of the human neuropeptide Y1 receptor in pain management [52].

The internal distress caused by either toxin- or RNAi-mediated targeting of core cellular processes or pathogenic colonization-induced intestinal bloating promotes associative learned avoidance *via* KGB-1 and NPR-1 [18, 42]. Our findings on the retrieval of ccBA- and ccDA-specific behaviors by the stress-associated olfactory cues also indicate learned avoidance. The involvement of similar signaling pathways suggests that volatiles elicit similar behavioral defenses to pathogens and soluble toxins, through the perception of internal distress. Moreover, repeated stress exposures with inter-trial “rest” intervals using spaced training stabilized the newly learned behaviors after the 2-h recovery. This observation is in agreement with the general nature of spaced training [43] as well as its stabilizing impact on *C. elegans* long term memory [44, 45]. The elimination of adaptation and habituation by employing food, spaced training, and recovery, as well as the association of stress-specific avoidant behaviors with the specific olfactory cues, strongly argues for the formation of associative memory. Besides the apparent associations with diluted BA and DA governing the chemotaxis and odor choice, it is likely that further associations with the bacterial cues present during preconditioning or spaced training also play a role in food leaving. Although our and the abovementioned studies [18, 42] shed light mainly on non-neuronal events that lead to aversion, the neuronal circuits controlling behavior and the mechanisms driving learning, memory formation, and retrieval are intriguing directions of further systematic work in the different experimental paradigms of toxic and pathogen stress.

The elicitation of opposing, avoidant, and tolerant behaviors by DA and BA olfactory cues after preconditioning demonstrates that the absence or presence of adequate cytoprotective responses at the time of stress is a critical regulator of future behavioral “fight-or-flight” decisions to anticipated stress. These findings suggest that the internal experiences during stress are integrated into respective memories of danger and protection depicted in Fig. 9. The increased preference of DA over BA after ccBA preconditioning suggests that the natural valence of BA is integrated with the cost to maintain it, probably through the experiences of toxic stress and induced defense. Such a representation allows individuals to consider whether an investment of resources for self-protection is needed or not, to obtain food.

The “fight-or-flight” response is an evolutionarily conserved adaptive response to stress, originally coined for the vertebrate neuroendocrine system [2]. Recent studies in *C. elegans* showed the co-occurrence of behavioral “flight” responses with molecular stress and immune “fight” responses combating stress [18–20]. Our studies in addition to an independent confirmation of the co-occurrence of these responses, reveal a regulatory link between intracellular cytoprotective responses and behavioral tolerance, suggesting a coordinated action of the fight and the flight responses to combat toxic stress. Beyond ensuring survival, the maintenance of cellular homeostasis by stress responses equips nematodes with behavioral tolerance to approach real and anticipated stressful locations. Further, temporary avoidance in stresses that overwhelm molecular defenses allows the restoration of physiological homeostasis and the expression of cytoprotective genes. Genetically weakened or absent molecular defenses might hinder access to resources by reinforcing avoidant behavior. Our work implies that memories of past stresses accompanied by insufficient cellular defenses may condition to avoidance. Avoidant behaviors are characteristic to various human mental disorders, such as phobias, panic attacks, post-traumatic stress disorder, and eating disorders. These diseases are accompanied by intense physical sensations of stress, overwhelming fear, and emotions to jettison or to avoid perceived danger, which happen in response to specific or unidentified sensory cues [7, 53]. The foundations of stress and detoxification responses and learning are conserved between nematodes and humans [1, 3, 5, 12]. Thus, it might be conceivable that unconscious memories of prior stressful physical experiences govern emotions and behaviors in response to sensory cues.

## Conclusions

This study shows how organisms ensure optimal self-protection during environmental stress by coordinating physiological and behavioral defenses. Specifically, our findings reveal a critical role of peripheral tissue defenses in regulating learned behavioral avoidance via the activation of conserved cellular stress responses. The mechanism depicted here enables animals to anticipate adverse conditions by retrieving stress memories and tailor their behavioral decisions depending on their past physiological response to the stressor. Whether such cellular memories might shape mammalian behavior is subject of future studies.

## Methods

### Materials

The reagents benzaldehyde, diacetyl, methyl-salicylate, and paraquat-dichloride hydrate were obtained from Sigma-Aldrich. ccBA and ccDA abbreviate undiluted benzaldehyde and diacetyl, respectively. All other chemicals were obtained from Sigma or Fluka, if not otherwise mentioned.

### *C. elegans* strains and maintenance

The following strains used were provided by the Caenorhabditis Genetics Center: N2 (Bristol) wild type; KU25 [*pmk-1(km25)* IV]; KU21 [*kgb-1(km21)* IV]; KQ1564 [*sgk-1(ft15)* X]; VC8 [*jnk-1(gk7)* IV]; CX4148 [*npr-1(ky13)* X]; TJ356 [*daf-16p::daf-16a/b::GFP + rol-6(su1006)*]; TJ375 [*hsp-16.2p::gfp*]; CF1038 [*daf-16(mu86)*]; CY573 [*bvls5(cyp-35B1p::gfp +gcy-7p::gfp)*]; SJ4005 [*hsp-4p::gfp*]; CF1553 {muIs84[pAD76(*sod-3p::gfp*)]}. Further strains used were as follows: LD001 *Is007 [skn-1::gfp]* (Tibor Vellai, Eötvös Loránd University, Budapest, Hungary), MJCU017 *kIs17[gst-4::gfp*, *pDP#MM016B]X* (Johji Miwa, Chubu University, Kasugai, Japan), and LD1171 *Is003 [gcs-1p::gfp]* (T. Keith Blackwell, Harvard Medical School, Boston MA, USA). Strains were grown and maintained as previously described [54]. Animals were synchronized by allowing adults to lay eggs for 4 h. All experiments were performed using day 1 adults, except the SKN-1::GFP localization followed in L3 larvae.

### Food avoidance assay

Two hundred microliters of concentrated overnight cultures of OP50 bacteria was dropped onto the center of 6 cm NGM plates and kept at room temperature overnight. Fifty to 80 synchronous day 1 adults were washed twice with M9 buffer and dropped onto the OP50 lawn and were allowed to settle for 30 min or 15 min after odorant preconditioning (PC) and spaced training (ST) protocols. A drop of the given odorant was placed on a piece of parafilm in the middle of the OP50 lawn. Animals on or off the lawn were counted at the indicated times. Worms incapable to move or crawled off the agar surface were censored. Assays were run in triplicates. The aversion index was calculated as *N*_off_ /*N*_total_.

### Odorant preconditioning and spaced training

Preconditioning treatments were performed using the hanging drop method to prevent direct contact of undiluted volatiles with worms in the presence of a large bacterial lawn. Precisely, 1 μl and 4 μl drop of undiluted benzaldehyde (ccBA) or diacetyl (ccDA), respectively, was placed on the lid of 6 cm NGM plates seeded with OP50, containing a synchronous population of 200–300 young adults. The plate was sealed with parafilm to maintain a constant dose of volatile and worms were preconditioned for 4 h or for the times indicated in the figure legends. Spaced training protocol was designed as described [44, 45] employing four sequential one-hour exposures to hanging drops of 2 μl ccBA, 4 μl ccDA, or vehicle with inter-trial 10-min “rest” periods allowing the animals to settle during gentle washes in M9 buffer in 50-ml Falcon tubes.

### Paralysis assay

Determination of paralysis was carried out by using approximately 25–40 worms per plate in triplicates in 3 cm NGM plates. ccBA and ccDA doses were used and expressed proportionally to the volumes used in the 6-cm plates. Paralyzed worms were scored by lack of movement in response to a gentle drop of the plate to the surface. Animals that crawled off the agar surface were censored.

### Survival assay

Determination of survival was carried out by using approximately 25–40 worms per plate in triplicates in 3 cm NGM plates. ccBA and ccDA doses were used and expressed proportionally to the volumes used in the 6-cm plates. Worms were scored 14 h after the end of toxic odorant exposures by tapping with a platinum worm pick. Animals that crawled off the agar surface were censored.

### RNA interference

The following HT115(DE3) *E. coli* dsRNA producing strains were used in the study: *daf-16* (Source BioScience, Notthingam, UK), *skn-1* (T. Keith Blackwell, Harvard Medical School, Boston, MA, USA), *wdr-23* (Keith P. Choe, University of Florida, Gainesville, FL, USA) and *hsp-*90 (Eileen Devaney, University of Glasgow, UK). RNAi treatments were performed as previously described [55]. RNAi feeding clones were grown overnight in LB medium containing 100 μg/ml ampicillin. Briefly, worms were grown on plates seeded with *E. coli* HT115 strains harboring the L4440 empty vector (EV) control and specific RNAi vectors, respectively, from hatching.

### Stereo and fluorescence microscopy

Photographs of animals on food leaving and on survival plates were carried out by an Olympus SZ61-Tr stereomicroscope with a Greenough optical system, under dark-field illumination with 0.67–3.5× magnifications and a CAM-EP50 5Mpx Camera. Analysis and quantification of fluorescence were carried out as previously described [16], with modifications. After treatments, at least 20 worms per condition were picked individually and immobilized by 20 mM NaN3 washed in M9 buffer onto a 2% agarose pad. Microscopic examination was carried out on a NIKON Eclipse E400 type fluorescence microscope linked to a Diagnostic Instruments SPOT 500 camera in case of TJ356, TJ375, CY573, MJCU017, LD1171, SJ4005, and CF1553 strains and OLYMPUS CKX53 Fluorescence microscope, OLYMPUS DP74 Cooled color camera in case of LD001 strain, using green fluorescent filters. Images are representatives of at least three independent experiments, except Fig. S3. Fluorescence intensity measurements were quantified with ImageJ. Visualization of SKN-1::GFP nuclear punctae were carried out by the OLYMPUS CellSens v2.3 Imaging software.

### Chemotaxis assay

Chemotaxis experiments were carried out as previously described [10] with modifications. Briefly, a synchronous population of young adults was washed twice in M9 buffer, then 80–100 worms were placed in the middle of a 10 cm CTX assay plate containing 1–1 μl of the odorant and vehicle at opposite sides of the plate without anesthetics. Assays were run in triplicates. The distribution of worms was determined after 30 min. Chemotaxis index was calculated as (# on odor) − (# on vehicle)/(total # on plate).

### Odor preference assay

Odor preference was carried out in standard CTX plates. 80–100 naive and preconditioned young adults were washed twice in M9 buffer and dropped into the middle of the assay plate containing 1–1 μl of the odorants at the opposite sides. Worms were allowed to migrate for 50 min, then counted in the 1 cm drawn circle around the respective odorants. Assays were run in triplicates. Data are expressed as the choice index given in the figure legends.

### Statistical analysis

Kaplan–Meier log-rank tests using the program IBM SPSS Statistics were carried out to evaluate toxicity assays. Food avoidance and chemotaxis assays were examined by one-way ANOVA with Fisher’s LSD post hoc test. Odor preference assays were analyzed by two-way ANOVA with Fisher’s LSD post hoc test after evaluation of normal distribution significance by the Shapiro-Wilk test. Significance in fluorescence intensity was calculated by unpaired Student’s *t* test following evaluation of normal distribution significance by the Kolmogorov-Smirnov test. One-way ANOVA with Fisher’s LSD post hoc tests, Shapiro-Wilk and Kolmogorov-Smirnov tests, and unpaired Student’s *t* test were carried out using IBM SPSS Statistics, while two-way ANOVA with Fisher’s LSD post hoc tests were performed with STATISTICA. Data were expressed as mean ± standard error of the mean (SEM). Statistical levels of significance are shown in the figures as follows: n.s., not significant; *p < 0.05; **p < 0.01; ***p < 0.001.

## Supplementary Information

**Additional file 1: Supplementary Methods.** Supplementary Methods – Kinetic Chemotaxis, Motility and Thermotolerance Assay. **Fig. S1.** Undiluted BA and DA on kinetic chemotaxis and thermotolerance. **Fig. S2.** Odor preconditioning-induced motility and aversion. **Fig. S3.** ccBA on DAF-16 translocation and expression of various stress reporters. **Fig. S4.** Effect of *daf-16* and *skn-1* RNAi on ccBA survival. **Fig. S5.** Toxicity and food aversion by undiluted methyl-salicylate. **Fig. S6** ccBA effect on food avoidance of *sgk-1* and *pmk-1* mutants.

**Additional file 2.** : Raw data and statistics to Figs. 1e, f and S1d and fluorescence data to Fig. 3f.

## Data Availability

All data generated or analyzed during this study are included in this published article (and its supplementary information files).
