# Supplementary material for: Toxic stress-specific cytoprotective responses regulate learned behavioral decisions in C. elegans
Source: BMC Biol. 2021 Feb 9;19:26. doi: 10.1186/s12915-021-00956-y (PMC7874617; doi:10.1186/s12915-021-00956-y)
Supplement: Supplementary file 1 — Additional file 1: Supplementary Methods. Supplementary Methods – Kinetic Chemotaxis, Motility and Thermotolerance Assay. Fig. S1. Undiluted BA and DA on kinetic chemotaxis and thermotolerance. Fig. S2. Odor preconditioning-induced motility and aversion. Fig. S3. ccBA on DAF-16 translocation and expression of various stress reporters. Fig. S4. Effect of daf-16 and skn-1 RNAi on ccBA survival. Fig. S5. Toxicity and food aversion by undiluted methyl-salicylate. Fig. S6 ccBA effect on food avoidance of sgk-1 and pmk-1 mutants. [file 12915_2021_956_MOESM1_ESM.pdf]

Toxic stress-specific cytoprotective responses regulate learned behavioral decisions in *C. elegans*

Supporting Information

Gábor Hajdú, Eszter Gecse, István Taisz[#], István Móra & Csaba Sőti*

Department of Molecular Biology, Semmelweis University, Budapest, Hungary

[#]*Current address: MRC Laboratory of Molecular Biology, Neurobiology Division, Cambridge, UK*

Content:

Supplementary Methods

Supplementary Figures S1-S6

***Corresponding author.** Department of Molecular Biology, Semmelweis University, P.O.Box 2, Budapest, H-1428, Hungary. Tel.: + 36 1 4591500 extn. 60130; Fax: + 36 1 4591500 extn. 60141; E-mail: soti.csaba@med.semmelweis-univ.hu

Supplementary Methods

Kinetic chemotaxis assay

In kinetic chemotaxis, 10 cm rectangular CTX assay plates were streaked at each centimeter to measure the weighted distribution of worms. Odors and vehicle were employed at opposite sides of the plates, as 3 spots of 3-3 µl ccBA and 6-6 µl ccDA (corresponding to 1 µl ccBA and 2 µl ccDA on 6 cm food leaving plates) without anesthetics. A synchronous population of young adults was washed twice in M9 buffer, then 80-100 worms were placed in the middle of the plate and counted at indicated time points. Assays were run in triplicates. The Weighted Chemotaxis Index (WCI) was calculated as previously described (*29*).

Motility assay

Motility was characterized by counting the body bends for 1 minute using 10-15 animals in each condition per assay. After measuring baseline motility on an OP50-seeded NGM agar plate, a toxic dose of odor hanging drop was placed on the lid and motility was measured at the indicated time points. To mimic the conditions of food leaving after preconditioning, after the 240-minute exposure animals were picked and placed onto fresh NGM plates and motility was measured after a 2-minute settling time.

Thermotolerance measurement

The thermotolerance assay was carried out at 35°C, respectively, by using approximately 25-40 worms per plate in triplicates on 6 cm NGM plates. Paralyzed worms were scored by lack of movement in response to a gentle drop of the plate to the surface. Animals that crawled off the agar surface were censored.

Supplementary Figures and Legends

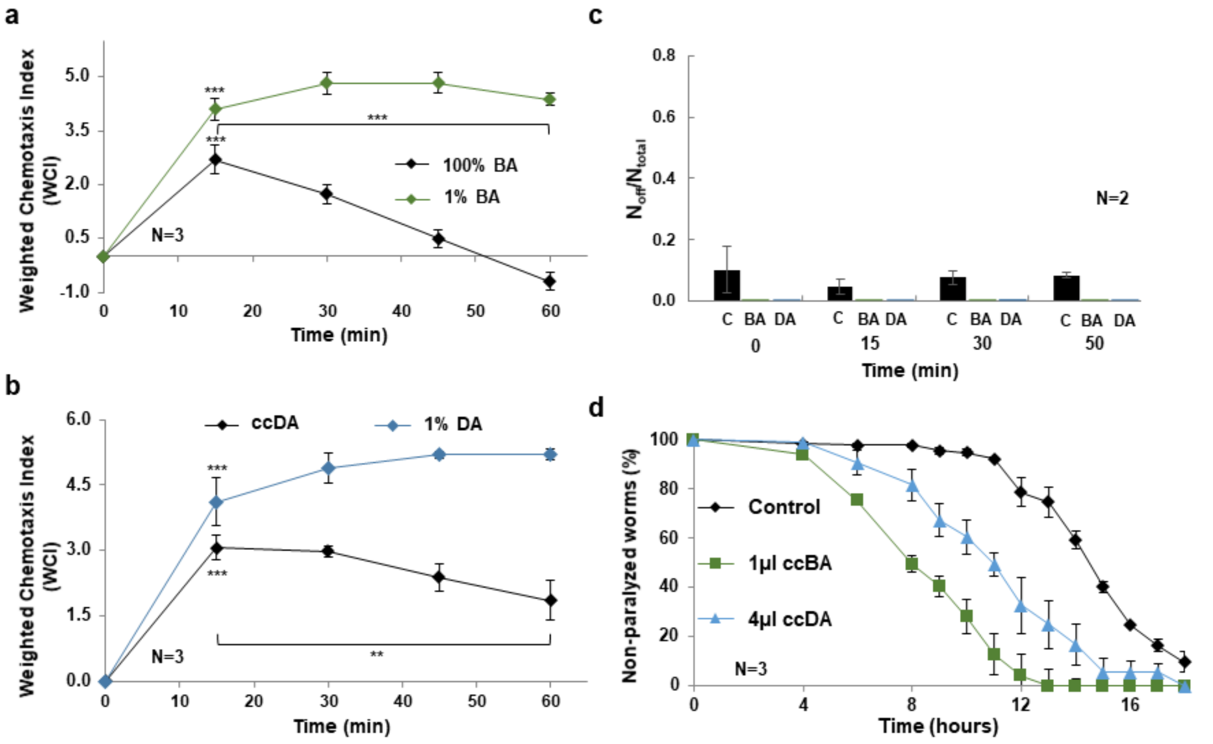

Fig. S1. Undiluted benzaldehyde (ccBA) and diacetyl (ccDA) induce behavioral and physiological alterations characteristic to toxicity, Related to Fig. 1.

a, b Undiluted, 100% BA (**a**) and DA (**b**) trigger initial attraction that diminishes over time as opposed to sustained attraction to diluted 1% odors. Odor sources were placed in the positive side in three drops (see Methods) opposite to three drops of vehicle. **c** Lawn avoidance behaviors in the presence of ethanol vehicle (C), or diluted 1 % BA and 1 % DA. **d** Continuous exposure to 1 µl ccBA and 4 µl ccDA impairs thermotolerance, the resistance to heat stress. Mean durations of heat shock that induced 50% paralysis by log rank (Mantel-Cox) test were as follows: 14.46 ± 0.23 hours for vehicle treated control, 10.74 ± 0.42 hours for ccBA-exposed (p=0.0001 compared to control), 12.45 ± 0.43 hours for ccDA-exposed (p=0.011 compared to control). Error bars represent mean +/− SEM. N = number of independent experiments. p values

of a, b, and c were obtained by one-way ANOVA with Fisher's LSD post hoc test. *p < 0.05; **p < 0.01; ***p < 0.001.

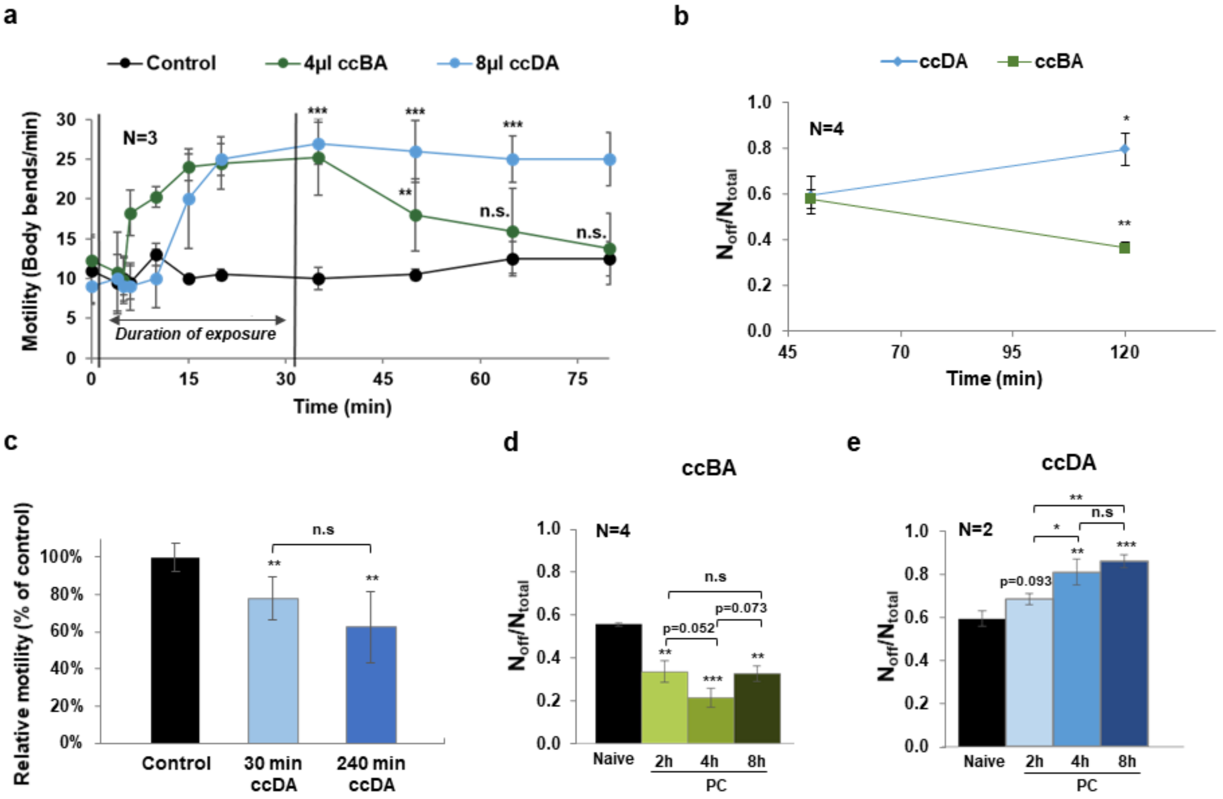

Fig. S2. Opposing effects of odor pre-exposure on odor induced behaviors, Related to Fig. 2.

a Motility assays show reversible *vs.* sustained elevation in locomotion in response to a 30-minute exposure to ccBA *vs.* ccDA. **b** Food aversion data showing that extended odor exposure to ccBA decreases, whereas that to ccDA further increases aversive behavior. **c** Decreased motility of animals exposed to 4 µl ccDA used in the food leaving assay. **d** ccBA-induced food avoidance as a function of duration of preconditioning exposure. **e** ccDA induced food avoidance as a function of duration of preconditioning exposure. Data are expressed as mean ± SEM. N = number of independent experiments. p values were obtained by one-way ANOVA with Fisher's LSD post hoc test. n.s.: not significant; *p < 0.05; **p < 0.01; ***p < 0.001.

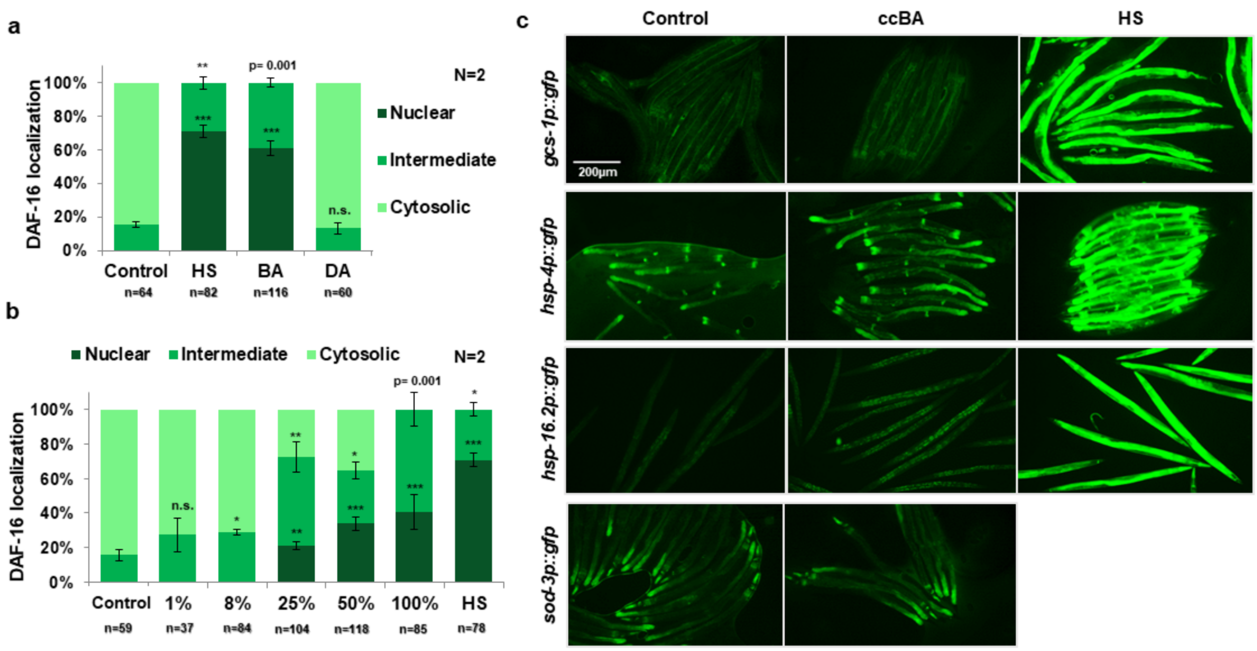

Fig. S3. Characteristics of DAF-16 nuclear translocation and specificity of ccBA-induced cytoprotective responses, Related to Fig. 3.

a ccBA induces nuclear translocation of DAF-16::GFP comparable to that upon heat shock (HS) (quantification of fluorescence intensities of the experiment from Fig. 3a). **b** Concentration-dependence of BA-induced DAF-16::GFP nuclear translocation. Animals were treated for 30 minutes with varios concentrations of BA in ethanol (control) in 1 µl total volume. Data are expressed as % of worms exhibiting the indicated DAF-16::GFP localization. Cytosolic refers to animals without nuclearly localized GFP signal, intermediate refers to animals with nuclear and cytosolic GFP and nuclear refers to animals with solely nuclear GFP signal. **c** Representative epifluorescent microscopic images of *hsp-16.2p::gfp*, *hsp-4p::gfp*, *gcs-1p::gfp* and *sod-3p::gfp* stress reporters upon a 4-hour exposure to 1 µl ccBA and a 4 hour 35ºC heat stress heat shock (HS) as a positive control. Please not the ccBA-induced increase of intestinal autofluorescence in the *hsp-16.2::gfp* animals. Data are expressed as mean of two independent experiments, n=number of evaluated animals.. p values were obtained by ANOVA with LSD post hoc test. n.s.: not significant; *p < 0.05; **p < 0.01; ***p < 0.001.

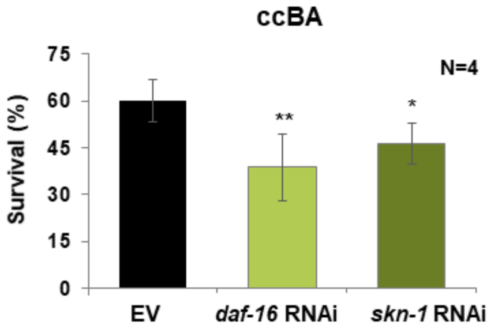

Fig. S4. RNAi silencing of *daf-16* and *skn-1* impaires survival upon ccBA exposure, Related to Fig. 4.

Survival of N2 worms fed by EV, *daf-16* RNAi and *skn-1* RNAi 14 hours after a 3-hour exposure to 8 µl ccBA. Error bars represent mean ± SEM, N = number of independent experiments. p values were obtained by ANOVA with Fisher's LSD post hoc test. n.s.: not significant; *p < 0.05; **p < 0.01.

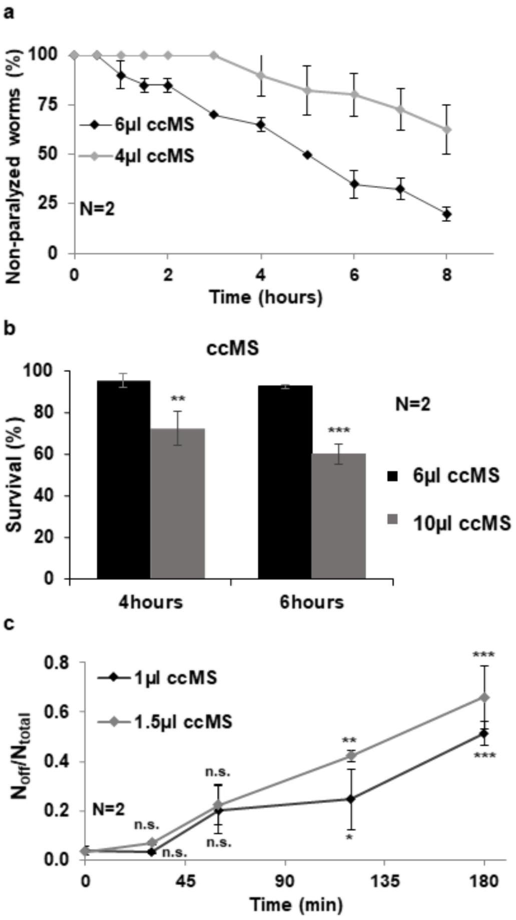

Fig. S5. Undiluted methyl-salicylate (ccMS) exposure causes toxicity and triggers food avoidance behavior, Related to Fig. 5.

a Paralysis curves of nematodes exposed to the indicated doses of ccMS using a hanging drop assay. **b** Survival of worms 14 hours after exposure to the indicated doses of ccMS for the indicated times. **c** Time-dependence of ccMS induced food avoidance behavior. Mean paralysis values by log rank (Mantel-Cox) test were as follows: 6.65 ± 0.32 hours for 4 µl ccMS, 4.72 ± 0.42 hours for 6 µl ccMS, p=0.001. Error bars represent mean ± SEM. N = number of independent experiments. p values were obtained by one-way ANOVA with Fisher's LSD post hoc test. n.s.: not significant; *$p < 0.05$, **$p<0.01$, ***$p<0.001$.

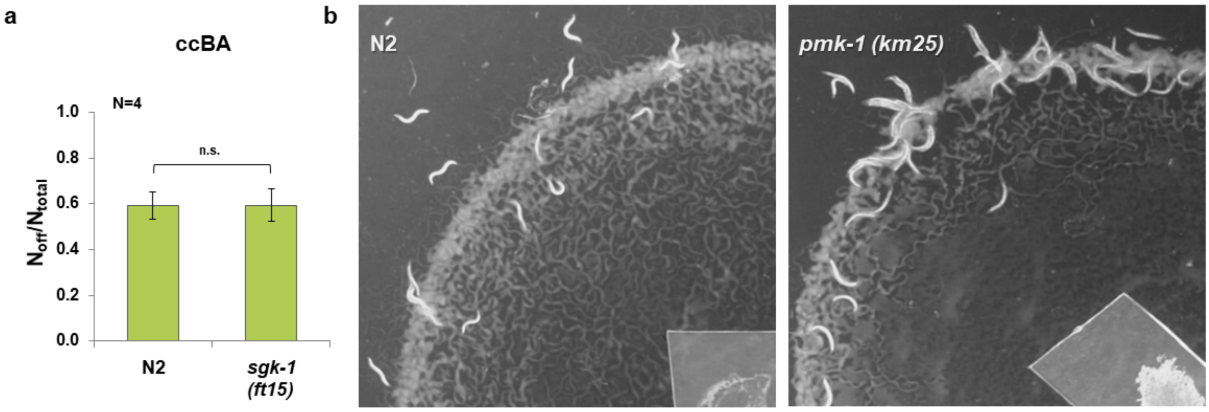

Fig. S6. ***sgk-1*** **mutants exhibits wild-type food leaving, whereas** ***pmk-1*** **mutants show extensive paralysis in response to ccBA exposure, Related to Fig 6.**

a Mutants defective in the non-stress-related serum glucocorticoid kinase 1 (*sgk-1)* show similar ccBA-induced food avoidance to that of N2. Error bars represent mean ± SEM. N = number of independent experiments. p values were obtained by one-way ANOVA with Fisher's LSD post hoc test. n.s.: not significant. **b** Robust paralysis and death of *pmk-1* mutants at the site of their locations compared to N2. Photographs were taken after a 30-minute exposure to 1 µl ccBA.
